# Integrative taxonomy and molecular phylogeny of three poorly known tintinnine ciliates, with the establishment of a new genus (Protista; Ciliophora; Oligotrichea)

**DOI:** 10.1186/s12862-021-01831-8

**Published:** 2021-06-09

**Authors:** Rui Wang, Yang Bai, Tao Hu, Dapeng Xu, Toshikazu Suzuki, Xiaozhong Hu

**Affiliations:** 1 College of Fisheries, & Key Laboratory of Mariculture, Ministry of Education, Ocean University of China, Qingdao, 266003 China; 2 Institute of Evolution & Marine Biodiversity, Ocean University of China, Qingdao, 266003 China; 3 Laboratory of Protozoology, Key Laboratory of Ecology and Environmental Science in Guangdong Higher Education, South China Normal University, Guangzhou, 510631 China; 4 State Key Laboratory of Marine Environmental Science, Institute of Marine Microbes and Ecospheres, College of Ocean and Earth Sciences, Xiamen University, Xiamen, 361102 China; 5 Faculty of Fisheries, Nagasaki University, 1‑14 Bunkyo-machi, Nagasaki, 852‑8521 Japan

**Keywords:** Biodiversity, loricate ciliates, new combination, new genus, rDNA, Tintinnina

## Abstract

**Background:**

The taxonomic classification of the suborder Tintinnina Kofoid & Campbell, 1929, a species-rich group of planktonic ciliated protistans with a characteristic lorica, has long been ambiguous largely due to the lack of cytological and molecular data for most species. *Tintinnopsis* is the largest, most widespread, and most taxonomically complex genus within this group with about 170 species occurring in nearshore waters. Previous molecular phylogenetic studies have revealed that *Tintinnopsis* is polyphyletic.

**Results:**

Here we document the live morphology, infraciliature, gene sequences, and habitat characteristics of three poorly known tintinnine species, viz. *Tintinnopsis karajacensis* Brandt, 1896, *Tintinnopsis gracilis* Kofoid & Campbell, 1929, and *Tintinnopsis tocantinensis* Kofoid & Campbell, 1929, isolated from the coastal waters of China. Based on a unique cytological feature (i.e., an elongated ciliary tuft with densely arranged kinetids) in the former two species, *Antetintinnopsis* gen. nov. is erected with *Antetintinnopsis hemispiralis* (Yin, 1956) comb. nov. (original combination: *Tintinnopsis hemispiralis* Yin, 1956) designated as the type species. Moreover, *A. karajacensis* (Brandt, 1896) comb. nov. (original combination: *Tintinnopsis karajacensis* Brandt, 1896) and *A. gracilis* (Kofoid & Campbell, 1929) comb. nov. (original combination: *Tintinnopsis gracilis* Kofoid & Campbell, 1929) are placed in a highly supported clade that branches separately from *Tintinnopsis* clades in phylogenetic trees based on SSU rDNA and LSU rDNA sequence data, thus supporting the establishment of the new genus. One other species is assigned to *Antetintinnopsis* gen. nov., namely *A. subacuta* (Jörgensen, 1899) comb. nov. (original combination *Tintinnopsis subacuta* Jörgensen, 1899).

**Conclusions:**

The findings of the phylogenetic analyses support the assertion that cytological characters are taxonomically informative for tintinnines. This study also contributes to the broadening of our understanding of the tintinnine biodiversity and evolution.

**Supplementary Information:**

The online version contains supplementary material available at 10.1186/s12862-021-01831-8.

## Background

Ciliates (phylum Ciliophora Doflein, 1901) are unicellular, heterokaryotic eukaryotes occurring worldwide in a multitude of diverse habitats including freshwater, brackish, and marine aquatic environments, soil, or associated with animals or plants [1–9]. As the most speciose group among the phylum, most tintinnines (suborder Tintinnina Kofoid & Campbell, 1929) are ubiquitous in coastal and oceanic water bodies. Furthermore, they are of great interest to protozoologists working in the field of planktonic ecology for the following reasons: i. they are ideal models for examining the diversity and biogeography of protistans [10, 11]; ii. they are bioindicators of water quality status and hydrological circulation [12–16]; iii. they are consumers of algae (primary producers) and prey for medium-sized metazoans (e.g. fish larvae), and thus play an important role in the transfer of matter and energy between the microbial loop and classical food chain [17–19].

Tintinnines are characterized by the possession of a lorica, which may be simple (tube- or vase-shaped) or complex (irregular-shaped) [20]. Since the first tintinnine was originally described by Müller [21] under the name of *Trichoda inquilinus*, more than 1000 nominal species have been described based almost exclusively on their lorica characters [22–28]. Nevertheless, lorica-based approaches alone are now thought to be inadequate for species identification and discrimination, since the lorica could be polymorphic owing to the influence of environmental conditions or physiological state of the cell itself [23, 28]. Recently, a few tintinnine species were studied using integrative techniques (live observation, silver staining, electron microscopy, and gene sequencing), which resulted in new insights into their systematics [29–31]. There is increasing evidence of lorica plasticity and cryptic species diversity among tintinnines [24, 25, 32–34]. Using tintinnines as a model, Santoferrara et al. [35] updated procedures for species identification of loricate protists based on integrative studies of their morphology (cellular and lorica), gene sequence data, and ecology.

*Tintinnopsis* was established by Stein [36] with *T. beroidea* Stein, 1867 as type species and was later revised by Jörgensen [37] and Kofoid & Campbell [26]. Currently, there are about 170 nominal species of *Tintinnopsis* which renders it the largest genus within Tintinnina [38]. The genus is characterized by the possession of an agglutinated and hard lorica. However, species delimitation remains difficult because many species overlap in terms of lorica size and shape. To date, cellular features have been investigated in detail for only 17 species of *Tintinnopsis* and SSU rDNA sequences are available for only 22 species [39–50]. Furthermore, combined data of cytological features and SSU rDNA sequences are available for only ten of these species. Previous molecular phylogenetic studies have revealed that *Tintinnopsis* is polyphyletic [51, 52], most of its members being distributed among at least eleven lineages with some species clustering with taxa that have either a sparsely agglomerated lorica (*Leprotintinnus*, *Rhizodomus*, *Stylicauda*) or a particle-free lorica (*Climacocylis*, *Helicostomella*) [52]. These stable and well-supported clades indicated that these unique and ambiguous *Tintinnopsis*-like species may belong to several distinct genera and families as indicated by their divergent cell features, lorica ultrastructure, and, especially, synapomorphies of the somatic ciliary pattern, which have been confirmed as key diagnostic trait at genus level in other well-known genera [29, 52].

In the current study, we redescribed three tintinnine ciliates, viz. *Tintinnopsis karajacensis* Brandt, 1896, *T. gracilis* Kofoid & Campbell, 1929, and *T. tocantinensis* Kofoid & Campbell, 1929, from coastal waters of China. The lorica and cellular morphology of the former two species were investigated based on live and protargol-stained specimens for the first time. Based on its unique somatic ciliary pattern, *Antetintinnopsis* gen. nov. is established for the former two species as well *T. hemispiralis* Yin, 1956 and *T. subacuta* Jörgensen, 1899. Furthermore, the SSU rDNA and LSU rDNA sequences of the three species were characterized and analyzed to determine their phylogenetic positions within Tintinnina.

The ZooBank registration number of the present work is urn:lsid:zoobank.org:pub:3543CEC0-2490-416B-9120-9656645FE3A4.

## Results

### Taxonomy

Class Oligotrichea Bütschli, 1887.

Order Choreotrichida Small & Lynn, 1985.

Suborder Tintinnina Kofoid & Campbell, 1929.

New genus ***Antetintinnopsis* gen. nov.**

*Diagnosis.* Lorica cylindrical, hard with agglutinated particles, only anterior end open. Somatic ciliary pattern complex with an extremely long ciliary tuft derived from densely arranged kinetids in middle portion of ventral kinety. Posterior kinety below left ciliary field.

*ZooBank registration number*. FE8DAFDA-DE2C-464A-9421-93B50FF5320D.

*Type species*. *Antetintinnopsis hemispiralis* (Brandt, 1896) comb. nov.

*Species assignable*. *Antetintinnopsis gracilis* (Kofoid & Campbell, 1929) comb. nov. (original combination: *Tintinnopsis gracilis* Kofoid & Campbell, 1929); *Antetintinnopsis hemispiralis* (Yin, 1956) comb. nov. (original combination: *Tintinnopsis hemispiralis* Yin, 1956); *Antetintinnopsis karajacensis* (Brandt, 1896) comb. nov. (original combination: *Tintinnopsis karajacensis* Brandt, 1896); and *Antetintinnopsis subacuta* (Jörgensen, 1899) comb. nov. (original combination: *Tintinnopsis subacuta* Jörgensen, 1899).

*Etymology*. The genus name *Antetintinnopsis* is a composite of the Latin prefix *ante*- (“before in place or time”) and the genus name *Tintinnopsis*, indicating that the new genus is similar to *Tintinnopsis* in lorica features but differs in somatic ciliary pattern. Feminine gender.

### *Antetintinnopsis karajacensis* (Brandt, 1896) comb. nov. (Figs. 1, 2, 3; Table 1)

**Fig. 1:**
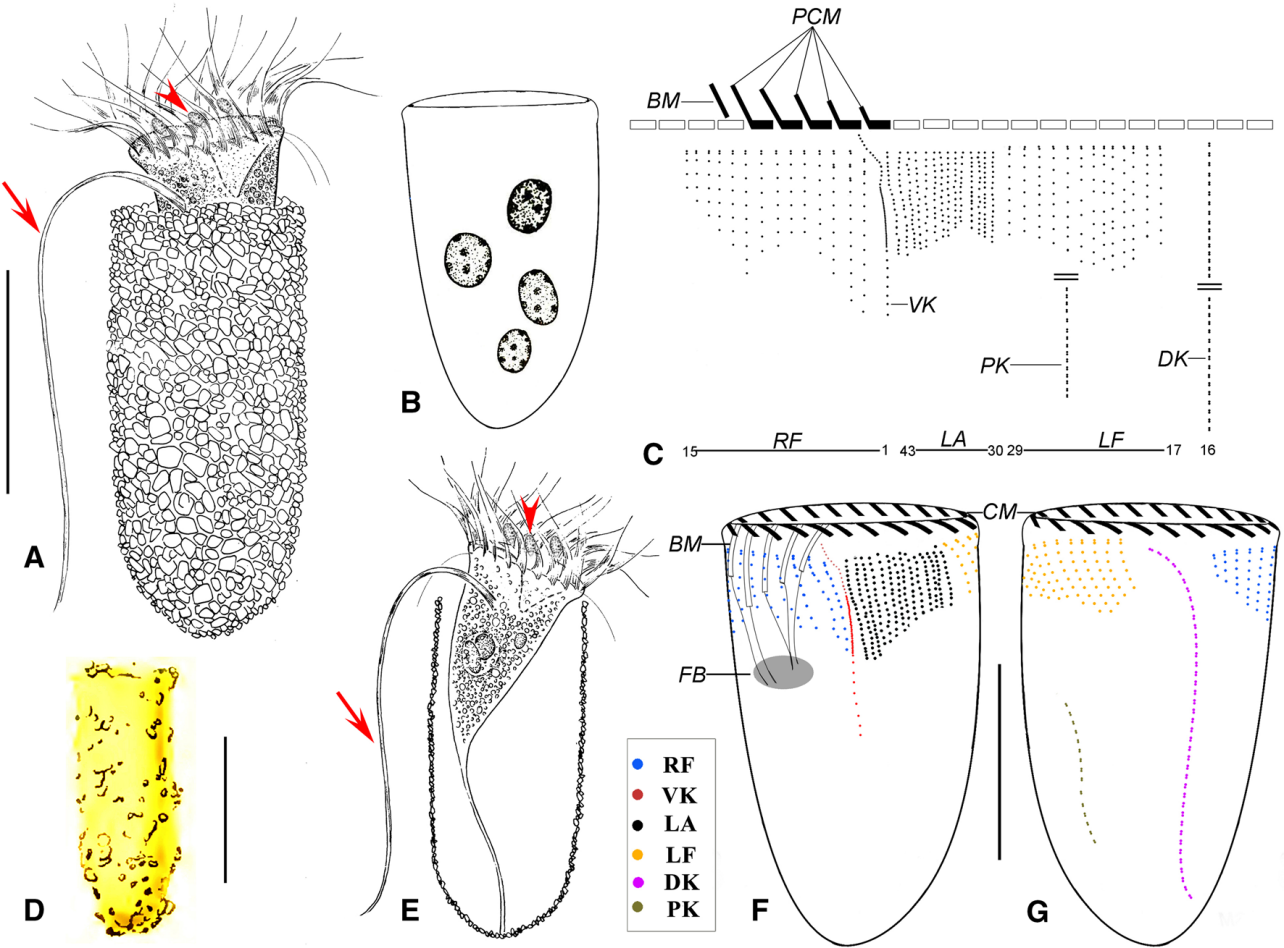
Drawing of *Antetintinnopsis karajacensis* comb. nov. in vivo (**A**, **D**–**E**) and after protargol staining (**B**–**C**, **F**–**G**). **A** Ventral view of a representative specimen, arrowhead and arrow denote the tentaculoids and elongated ventral ciliary tuft, respectively. **B** Macronucleus composed of four nodules. **C** Kinetal map of a morphostatic specimen. **D** From Brandt [56]. **E** Ventral view of the cell through the lorica, arrowhead shows the elongated ventral ciliary tuft. Ciliary pattern of ventral (**F**) and dorsal (**G**) side of a representative individual. BM, buccal membranelle; CM, collar membranelles; DK, dorsal kinety; FB, fiber bundles; LA, lateral ciliary field; LF, left ciliary field; PCM, prolonged collar membranelles; PK, posterior kinety; RF, right ciliary field; VK, ventral kinety. Scale bars = 40 μm

**Fig. 2:**
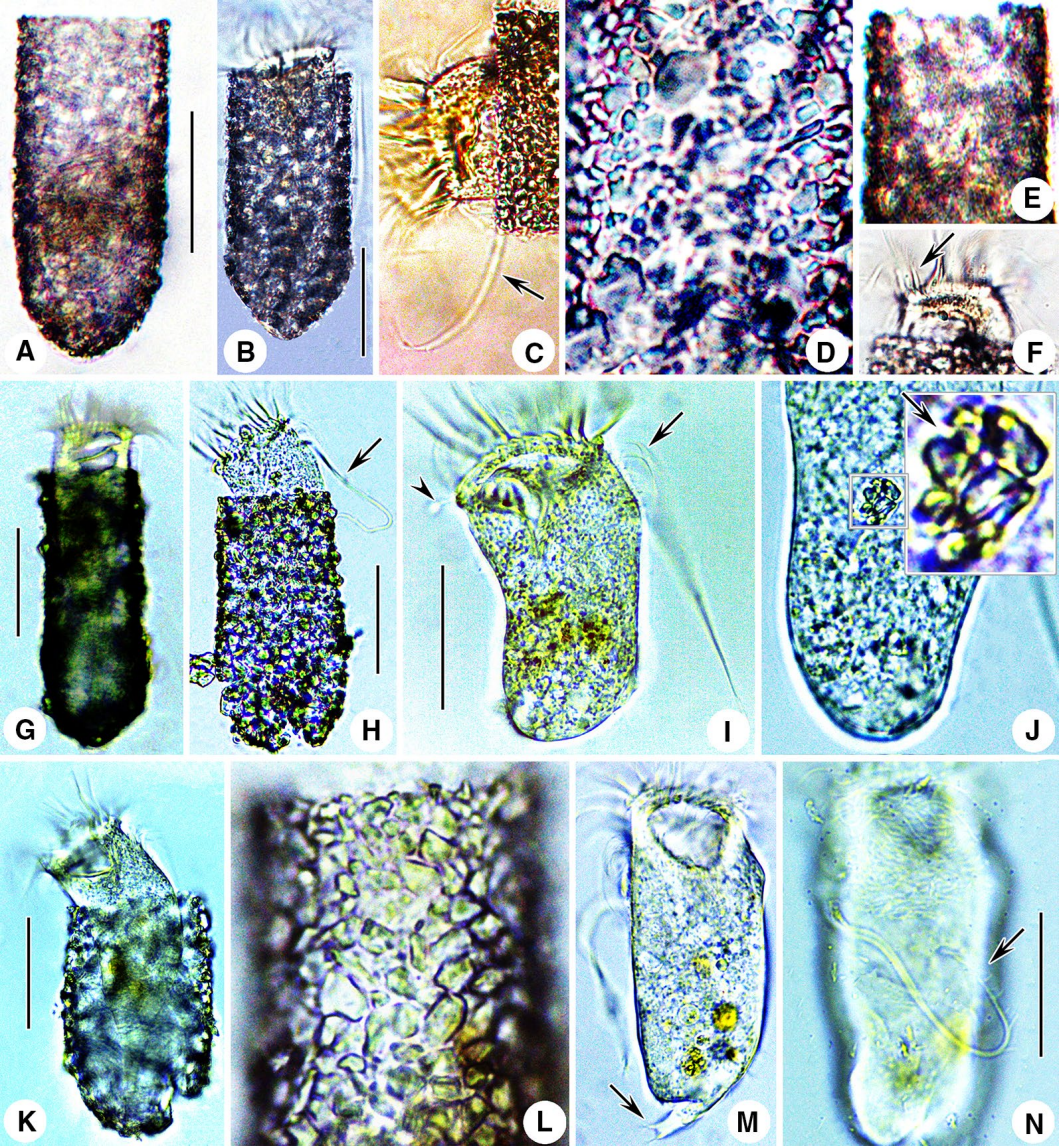
Photomicrographs of the Haikou population of *Antetintinnopsis karajacensis* comb. nov. in vivo (**A**–**F**). **A**, **B** Lateral views of two representative individuals showing the overall shape of lorica. **C** Anterior portion of a cell proper, arrow shows the elongated ciliary tuft. **D**, **E** Lorica wall with mineral particles. **F** Pin-shaped tentaculoids (arrow). **G**–**J** Photomicrographs of the Beihai population of *A. karajacensis* comb. nov. from live specimens. **G**, **H** Lateral views of different individuals showing variations of lorica shape and size, arrowhead denotes the elongated ciliary tuft. **I** Cell proper out of the lorica, arrow and arrowhead indicate the elongated cilium of left ciliary field and pin-shaped tentaculoids, respectively. **J** Portion of cell proper, showing amplification of the particles (arrow) within the cytoplasm. **K**–**N** Photomicrographs of the Zhoushan population of *A. karajacensis* comb. nov. from living specimens. **K** Lateral view showing cell in the lorica. **L** Details of the surface of lorica. **M** Fully extended cell, arrow denotes the branched peduncle. **N** Dorsal view of a cell proper, arrow indicates the bifurcation at the end of the elongated ciliary tuft. Scale bars = 40 μm (**A**, **B**, **G**, **H**, **I**, **N**), 20 μm (**K**)

**Fig. 3:**
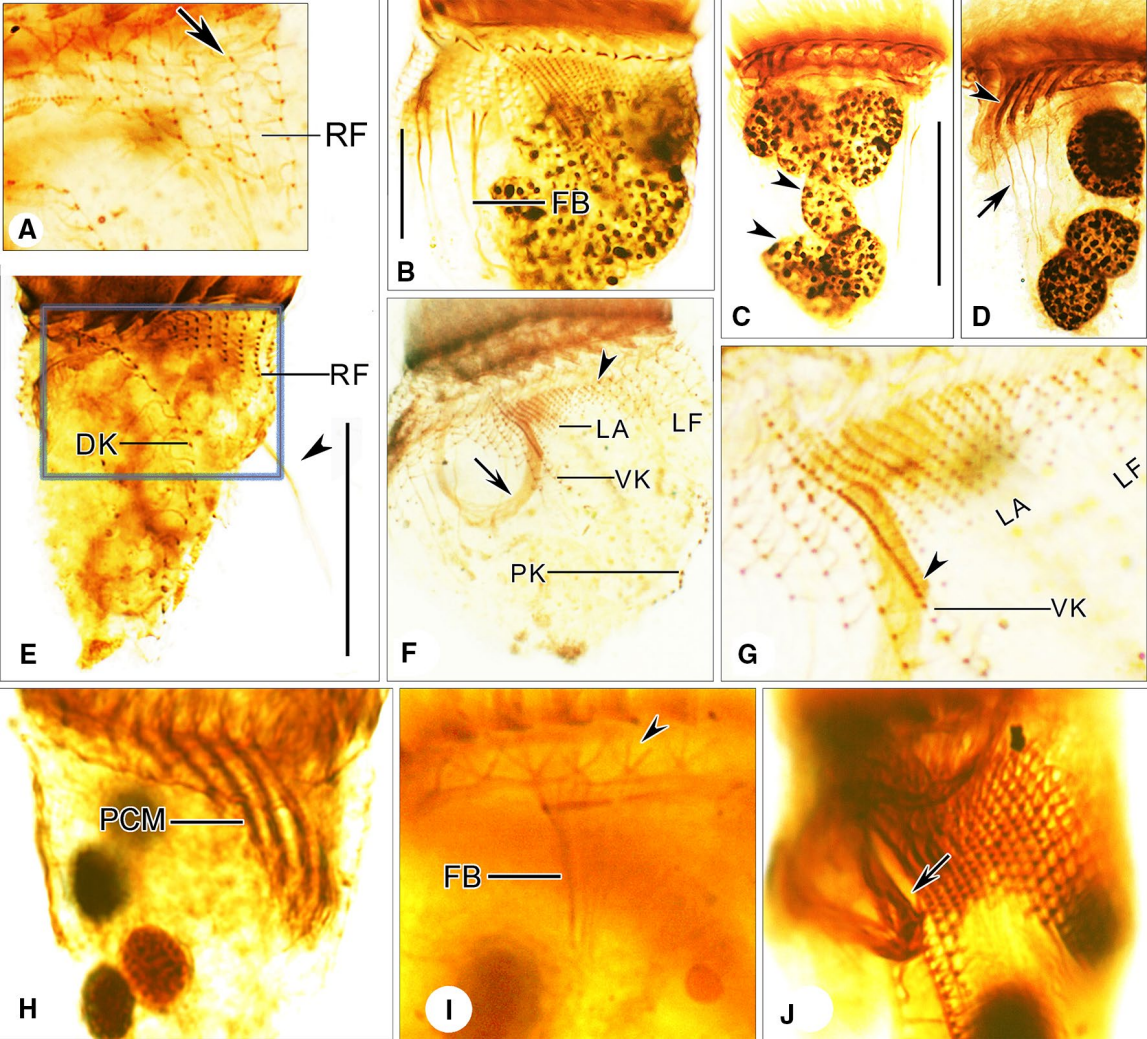
Photomicrographs of *Antetintinnopsis karajacensis* comb. nov. after protargol staining (**A**–**J**). **A**–**G** Specimens from Haikou. **A **Details of dorsal side showing the right ciliary field, arrow shows the anterior dikinetids. **B**–**D** Ventral views of representative individuals, arrowheads in (**C**) mark the macronuclear nodules; arrowhead and arrow in (**D**) mark the prolonged collar membranelles and fibers bundles, respectively. **E** Dorsal view of a representative individuals, arrowhead indicates the elongated ciliary tuft. **F** Ciliary patterns in ventral side of a crushed specimen, arrow marks elongated ciliary tuft and arrowhead indicates the anterior dikinetids in left ciliary field. **G** Details of ventral kinety and lateral ciliary field, arrowhead marks the densely arranged kinetosomes in the elongated ciliary tuft. **H**–**J** Specimens from Beihai, arrowhead in (**I**) denotes the bundles of argyrophilic fibers associated with distal end of each collar membranelle, arrow in (**J**) indicates the elongated ciliary tuft. DK, dorsal kinety; FB, fiber bundles; LA, lateral ciliary field; LF, left ciliary field; PCM, prolonged collar membranelles; PK, posterior kinety; RF, right ciliary field; VK, ventral kinety. Scale bars = 20 μm

**Table 1:** Morphometry of Haikou population (AK1), Beihai population (AK2), Zhoushan population (AK3) of *Antetintinnopsis karajacensis* comb. nov., *Antetintinnopsis gracilis* comb. nov. (AG), and *Tintinnopsis tocantinensis* (TT)

Characters^a^	Species	Min	Max	Mean	SD	CV	*n*
Lorica, total length^b^	AK1 AK2 AK3 AG TT	88 95 45 95 110	102 115 60 115 158	95.6 106.8 50.6 106.3 135.3	2.0 5.6 4.5 8.8 15.0	2.1 5.2 8.9 8.2 11.1	22 21 15 20 17
Lorica, width^b^	AK1 AK2 AK3 AG TT	41 35 35 39 30	48 44 42 55 60	43.6 38.3 38.5 47.3 48.2	1.5 1.7 2.0 4.0 9.8	3.4 4.4 5.2 8.5 20.3	22 21 15 20 17
Lorica, opening diameter^b^	AK1 AK2 AK3 AG TT	41 35 35 35 15	46 40 43 44 35	44.0 37.6 39.4 39.6 24.5	1.5 2.0 2.6 2.4 3.8	3.4 5.3 6.6 6.1 15.5	21 21 15 20 17
Lorica length: opening diameter, ratio^b^	AK1 AK2 AK3 AG TT	1.92 2.27 1.67 3.28 5.1	2.42 2.84 2.2 3.91 7.3	2.22 2.52 1.86 3.7 5.7	0.2 0.2 0.2 0.2 0.7	9.0 7.9 10.8 5.4 12.3	21 21 15 20 17
Cell proper, length	AK1 AK2 AK3 AG TT	50 53 37 70 42	72 75 58 85 75	61.5 63.4 44.5 77.0 55.8	5.4 6.6 7.8 4.3 8.9	8.8 10.4 17.5 5.6 16.0	20 18 14 18 17
Cell proper, width	AK1 AK2 AK3 AG TT	22 26 17 26 20	40 38 33 39 35	32.0 33.0 23.2 34.6 28.8	4.8 2.9 4.9 3.5 4.1	15.0 8.8 21.1 10.1 14.2	20 18 14 18 17
Macronuclear nodules, number	AK1 AK2 AK3 AG TT	2 2 3 7 2	8 8 7 12 2	4.6 4.4 4.7 9.8 2.0	1.6 1.3 1.2 1.4 0.0	34.8 29.5 25.5 14.3 0.0	18 17 13 16 13
Anterior macronucleus nodule, length	AK1 AK2 AK3 AG TT	9 10 6 7 8	16 18 13 12 23	12.6 14.5 9.5 8.7 13.8	2.1 1.7 1.9 1.5 4.3	16.7 11.7 20 17.2 31.2	18 17 13 16 13
Anterior macronucleus nodule, width	AK1 AK2 AK3 AG TT	6 7 4 8 7	14 14 9 10 19	9.8 9.2 6.8 9.3 11.8	1.8 1.7 1.3 1.1 1.8	18.4 18.5 19.1 11.8 15.3	18 17 13 16 13
Anterior cell end to anterior macronucleus nodule, distance	AK1 AK2 AK3 AG TT	6 7 5 11 9	12 13 14 22 24	8.2 9.4 8.4 16.3 16.5	1.7 2.0 2.5 2.8 3.2	20.7 21.3 29.8 17.2 19.4	18 17 13 16 13
Ventral kinety, length	AK1 AK2 AK3 AG TT	22 19 15 30 15	38 32 24 45 23	28.2 27.3 20.2 34.4 17.3	3.3 3.2 2.8 3.0 2.2	11.7 11.7 13.5 8.7 12.7	17 16 13 16 15
Ventral kinety, distance to collar membranelles	AK1 AK2 AK3 AG TT	2 2 2 2 2	6 6 5 5 8	3.4 3.5 4.0 3.2 4.4	1.2 1.2 1.0 0.9 1.5	35.3 34.3 25.0 28.1 34.1	17 16 13 16 15
Right ciliary field, number of kineties	AK1 AK2 AK3 AG TT	11 10 10 10 6	13 12 12 13 8	11.4 11.0 10.9 11.5 7.0	0.7 0.6 0.7 0.9 0.5	6.1 5.5 6.4 7.8 7.1	17 17 12 15 15
Kinety 1 in right ciliary field, length	AK1 AK2 AK3 AG TT	12 12 9 22 11	17 16 14 41 21	14.7 15.1 12.8 29.5 15.2	2.0 2.3 2.2 4.2 3.0	13.6 15.2 17.2 14.2 19.7	17 17 12 15 15
Kinety 1 in right ciliary field, distance to collar membranelles	AK1 AK2 AK3 AG TT	5 5 4 3 5	9 10 8 8 10	6.5 7.0 5.6 5.4 7.6	1.1 1.6 1.3 1.2 1.2	16.9 22.9 23.2 22.2 15.8	17 17 12 15 15
Kinety 1 in right ciliary field, number of kinetids	AK1 AK2 AK3 AG TT	10 9 7 10 10	14 15 12 16 17	12.0 13.1 10.3 13.2 13.6	1.3 1.5 1.4 1.7 1.7	10.8 11.5 13.6 12.9 12.5	17 17 12 15 15
Kinety *n* in right ciliary field, length	AK1 AK2 AK3 AG TT	2 2 2 3 7	5 6 9 9 12	2.8 3.1 4.1 6.2 9.7	0.8 0.9 1.3 1.2 1.1	28.6 29.0 31.7 19.4 11.3	17 17 12 15 15
Kinety *n* in right ciliary field, number of kinetids	AK1 AK2 AK3 AG TT	2 2 2 4 6	4 4 6 7 10	2.5 2.9 3.6 5.4 8.5	0.8 0.7 1.1 0.9 1.0	32.0 24.1 30.6 16.6 11.8	17 17 12 15 15
Dorsal kinety, length	AK1 AK2 AK3 AG TT	50 34 31 65 32	73 62 52 85 60	58.4 48.9 40.8 72.5 48.5	6.4 9.1 5.8 7.5 7.2	10.9 18.6 14.2 10.3 14.8	17 15 13 15 14
Dorsal kinety, number of dikinetids	AK1 AK2 AK3 AG TT	25 25 16 30 22	43 35 32 50 48	35.2 28.3 25.7 39.4 31.3	5.3 3.4 4.3 5.7 6.4	15.1 12.0 16.7 14.5 20.4	17 15 13 15 14
Dorsal kinety, distance to collar membranelles	AK1 AK2 AK3 AG TT	4 5 3 6 2	10 12 8 15 7	6.2 7.7 5.3 9.6 4.8	1.3 1.4 1.2 1.9 1.1	21.0 18.2 22.6 19.8 22.9	17 15 13 15 14
Dorsal kinety, distance to right ciliary field	AK1 AK2 AK3 AG TT	5 6 5 8 3	11 13 12 19 8	7.3 8.2 6.4 12.6 5.2	1.2 1.3 1.3 1.5 0.9	16.4 15.9 20.3 11.9 17.3	17 15 13 15 14
Left ciliary field, number of kineties	AK1 AK2 AK3 AG TT	11 10 10 12 8	14 12 13 17 10	12.3 11.0 11.3 14.5 9.2	0.8 0.7 0.8 1.1 0.8	6.5 6.4 7.1 7.6 8.7	17 15 13 15 14
Kinety *n* in left ciliary field, distance to collar membranelles	AK1 AK2 AK3 AG TT	2 3 2 2 3	6 8 5 5 9	4.1 5.3 3.2 3.4 6.3	0.5 1.0 0.8 0.6 1.2	12.2 18.9 25.0 17.6 22.6	17 15 13 15 14
Kinety *n* in left ciliary field, length	AK1 AK2 AK3 AG TT	7 8 6 15 3	16 17 15 30 6	14.0 16.4 11.7 23.6 4.6	2.8 3.0 2.6 4.8 0.7	20.0 18.3 22.2 20.3 15.2	17 15 13 15 14
Kinety *n* in left ciliary field, number of kinetids	AK1 AK2 AK3 AG TT	6 7 6 11 2	12 15 13 15 5	9.1 11.7 8.6 12.6 3.7	2.0 2.5 1.7 1.3 0.7	22.0 21.3 19.8 10.3 18.9	17 15 13 15 14
Kinety 1 in left ciliary field, length	AK1 AK2 AK3 AG TT	7 7 6 17 8	15 16 15 25 12	11.2 11.5 10.5 22.1 9.8	2.6 2.5 2.6 2.7 1.2	23.2 21.7 24.8 12.2 12.2	17 15 13 15 14
Kinety 1 in left ciliary field, number of kinetids	AK1 AK2 AK3 AG TT	8 8 6 9 10	12 12 11 13 14	10.4 10.6 9.5 11.3 12.1	1.6 1.6 1.4 1.1 0.9	15.4 15.1 14.7 9.7 7.4	17 15 13 15 14
Lateral ciliary field, number of kineties	AK1 AK2 AK3 AG TT	15 13 13 14 8	18 16 17 20 11	16.3 14.7 15.0 16.3 9.0	0.8 1.0 1.1 1.7 0.8	5.0 7.0 7.3 10.4 9.1	15 15 12 14 13
Lateral ciliary field, width	AK1 AK2 AK3 AG TT	6 5 4 19 13	10 8 7 25 17	7.4 6.3 5.8 21.5 15.2	1.2 1.0 1.2 2.6 1.5	16.2 15.9 20.7 12.1 9.9	15 15 12 14 13
Kinety 1 in lateral ciliary field, distance to collar membranelles	AK1 AK2 AK3 AG TT	3 3 2 2 3	7 8 6 5 7	5.2 5.7 4.6 3.4 5.4	0.9 1.2 0.8 0.8 0.7	17.3 21.1 17.4 23.5 13.0	15 15 12 14 13
Kinety 1 in lateral ciliary field, length	AK1 AK2 AK3 AG TT	9 8 6 15 16	16 15 13 25 32	13.2 13.0 10.4 18.5 26.0	2.0 2.3 2.0 3.0 4.2	15.2 17.7 19.2 16.2 16.2	15 15 12 14 13
Posterior kinety, length	AK1 AK2 AK3 AG TT	15 16 12 17 12	23 25 20 32 28	18.4 19.7 16.3 25.3 18.6	2.2 2.3 1.9 5.6 5.3	12.0 11.7 11.7 22.1 28.5	13 10 10 15 12
Posterior kinety, number of dikinetids	AK1 AK2 AK3 AG TT	11 9 9 11 7	22 20 19 19 15	16.3 15.9 15.3 14.6 11.5	2.3 2.1 1.9 1.7 1.6	14.1 13.2 12.4 11.6 14.0	13 10 10 15 12
Posterior kinety, distance to collar membranelles	AK1 AK2 AK3 AG TT	12 15 9 19 18	28 32 19 41 48	21.2 23.4 16.1 28.5 30.2	4.7 4.9 3.6 5.8 8.4	22.2 21.0 22.4 20.4 27.8	13 10 10 15 12
Posterior kinety, distance to dorsal kinety	AK1 AK2 AK3 AG TT	14 16 12 15 18	27 29 22 26 32	20.2 23.9 17.8 19.7 27.4	4.4 4.6 3.7 4.2 4.9	21.8 19.2 20.8 21.3 17.9	13 10 10 15 12
Adoral zone of membranelles, diameter	AK1 AK2 AK3 AG TT	20 24 18 26 16	35 33 30 35 32	28.7 28.5 25.5 29.2 25.7	3.8 2.7 3.0 2.4 4.5	13.2 9.5 11.8 8.2 17.5	16 15 13 18 14
Collar membranelles, number^c^	AK1 AK2 AK3 AG TT	22 21 21 21 20	25 25 23 25 24	23.0 22.4 22.1 23.2 22.5	0.2 1.3 0.7 0.2 0.3	0.8 5.8 3.2 0.8 1.3	16 15 13 18 14
Prolonged collar membranelles, number	AK1 AK2 AK3 AG TT	4 4 4 4 3	4 4 4 4 3	4 4 4 4 3	0 0 0 0 0	0 0 0 0 0	16 15 13 18 14
Buccal membranelles, number	AK1 AK2 AK3 AG TT	1 1 1 1 1	1 1 1 1 1	1 1 1 1 1	0 0 0 0 0	0 0 0 0 0	16 14 11 17 13

Min, minimum; Max, maximum; Mean, arithmetic mean; SD, standard deviation; CV, coefficient of variation in %; *n*, number of individuals examined

^a^Data are based, if not stated otherwise, on protargol-stained specimens; ^b^measured from field materials in vivo; ^c^counted from protargol-stained or live materials. Measurements in μm

*Improved diagnosis based on original population and three Chinese populations*. Subcylindrical lorica 45–120 × 35–50 μm in size, with an opening diameter of 35–45 μm wide, posterior portion slightly rounded. Cell proper obconical when fully extended, size 40–80 × 15–30 μm in vivo. On average, four ellipsoidal macronuclear nodules. Ventral kinety usually commencing anteriorly to first right kinety. Right ciliary field with on average 11 kineties including the three leftmost kineties, each with two or three anterior dikinetids. Left and lateral ciliary field with about 11 kineties and 15 kineties, respectively. On average 16 dikinetids in posterior kinety and 32 dikinetids in dorsal kinety. About 23 collar membranelles, of which four extend into buccal cavity; one buccal membranelle.

*ZooBank registration number*. 31AE1A2D-7CED-4E41-B112-E359E48B4F47.

*Deposition of neotype and voucher specimens*. Haikou population (pop. 1): a protargol slide including the neotype specimen (Fig. 3A, F) was deposited in the Laboratory of Protozoology, Ocean University of China (registration number: WR2017110301-1); one additional protargol slide with voucher specimens was deposited in the same collection (registration number: WR2017110301-2). Beihai population (pop. 2): slides with protargol-stained voucher specimens were deposited in the Laboratory of Protozoology, South China Normal University (registration number: HT2018071924a, HT2018071924b). Zhoushan population (pop. 3): slides with protargol-stained voucher specimens were deposited in the Laboratory of Protozoology, South China Normal University (registration number: HT2018081435a, HT2018081435b).

*Description based on three Chinese populations.* Lorica subcylindrical, about 45–115 × 35–48 μm in size, posterior end slightly rounded; about 10% specimens slightly swollen at posterior. Lorica opening 35–46 μm in diameter (Figs. 1A, 2A, B, F). Ratio of lorica length to opening diameter 1.67–2.84: 1. Lorica wall densely agglutinated with inorganic particles such as sand grains (3–7 × 2–4 μm) (Figs. 1A, 2A, B, D, J, L).

Cell proper elongate-obconical, projecting conspicuously far beyond opening rim when fully extended, size about 40–80 × 15–35 μm in vivo and 37–75 × 22–40 μm after protargol staining (Figs. 1E, 2H, I, M). Posterior portion of cell proper forming a contractile peduncle about 20–50 μm long, which attaches to the bottom of lorica (Figs. 1E, 2M). Two to eight (on average four or five) ellipsoidal macronuclear nodules scattered in cytoplasm, each about 6–18 × 4–14 μm after protargol staining (Figs. 1B, 3D). Micronuclei insufficiently stained to be observed. Tentaculoids slender pin-shaped, about 4–8 μm long, located in outer portions of intermenmbranellar ridges (Figs. 1A, E, 2F, I). Accessory combs, contractile vacuole, cytopyge, and capsules not observed in either living or stained specimens. Cytoplasm colorless, with several food vacuoles up to 7 μm across containing ingested yellow microalgae (Fig. 2I, M). Locomotion by swimming slowly while rotating about main cell axis (speed not measured), cell retracting quickly into lorica with its contractile peduncle when disturbed (Fig. 2A).

Somatic ciliary pattern consisting of a ventral, dorsal, and posterior kinety as well as a right, left, and lateral ciliary field (Figs. 1C, F, G, 3A–J). Ventral kinety commencing about 3 μm posteriorly to collar membranelles and 4 μm anteriorly to firstly kinety of right ciliary field, which is comprised of three portions: i. anterior portion which is composed of about 14 monokinetids about 1 μm apart, each bearing a cilium that is 1 μm long after protargol staining; anterior of this part curving slightly rightwards at same level as commencement of first kinety of right ciliary field; ii. middle portion which extends longitudinally, consisting of extremely densely spaced kinetids each bearing a long cilium, that is no less than the cell length forming the ciliary tuft; iii. posterior portion which terminates at mid-body, composed of about eight widely spaced monokinetids 2–3 μm apart, each bearing a cilium that is 2–4 μm long in stained specimens (Figs. 1C, F, 2C, I, M, N, 3F, J). Right ciliary field comprising 10–13 kineties each of which commences about 6 μm posteriorly to collar membranelles except for the first kinety which starts about 7 μm posteriorly to collar membranelles; each kinety separated by about 3 μm from its neighboring kineties; length of kineties and number of kinetids in each kinety highly variable; each kinety monokinetidal with one anterior dikinetid, except for the three leftmost kineties each of which has two or three anterior dikinetids; cilia in right field about 5 μm long in vivo and 3–5 μm long after protargol staining, except for cilia of anterior dikinetids which are about 15–20 μm in vivo and about 15 μm after protargol staining (Figs. 1C, E, F, 2I, 3A, E, F). Dorsal kinety dikinetidal, commencing about 6 μm posteriorly to collar membranelles and separated from right and left ciliary fields by conspicuously broad unciliated stripes (about 7 μm wide); anterior and posterior portions curving rightward; length ranging from 31 μm to 73 μm and the number of dikinetids from 16 to 43; only posterior basal body of each dikinetid bears a cilium that is about 3 μm long after protargol staining (Figs. 1C, F, G, 3E). Left ciliary field separated from collar membranelles by about 4 μm long unciliated stripe; consisting of about 10–14 kineties that slightly decrease in length in clockwise direction (when viewed from apical aspect); each kinety with 6–12 widely spaced monokinetids and one anterior dikinetid; each basal body bears a cilium about 3 μm long except for the anterior basal body of each dikinetid, which has a cilium about 15 μm in vivo and 12 μm after protargol staining (Figs. 1C, F, G, 3F, G). Lateral ciliary field commencing about 5 μm posteriorly to collar membranelles, composed of on average 16 monokinetidal kineties, which generally increase in length in clockwise direction (when viewed from apical aspect), each separated from its neighboring kineties by a 1 μm gap. Posterior kinety usually commencing about 25 μm posteriorly to collar membranelles, curving rightwards with posterior portion parallel to that of dorsal kinety, about 19 μm long and composed of on average 15 dikinetids; only posterior basal body of each dikinetid bears a cilium that is about 3–5 μm long after protargol staining (Figs. 1C, F, G, 3B, F, G).

Adoral zone of membranelles closed, 20–35 μm in diameter after protargol staining, orthogonal to main cell axis in contracted specimens. About 21–25 collar membranelles, with cilia about 30 μm long in vivo. Polykinetids of collar membranelles extending obliquely across peristomial rim and thus forming a contorted pattern (Figs. 1A, 2I, M, 3I). Along with the single buccal membranelle, five prolonged collar membranelles in buccal cavity, successively elongated, polykinetids of which terminate 6–12 μm below apical end of cell (Figs. 1F, 2I). Two bundles of argyrophilic fibers associated with distal end of each collar membranelle, about 5 μm long, extending rightwards and leftwards and merging into neighboring fibers underneath membranellar zone (Fig. 3I). Further bundles of fibers that originating from proximal ends of prolonged collar membranelles and buccal membranelle, extending posteriorly, terminating in posterior portion of the cell. Endoral membrane commencing in dorsal portion of peristomial field and extending into buccal cavity, composed of a single row of monokinetids (Fig. 3A).

### *Antetintinnopsis gracilis* (Kofoid & Campbell, 1929) comb. nov. (Figs. 4, 5; Table 1)

**Fig. 4:**
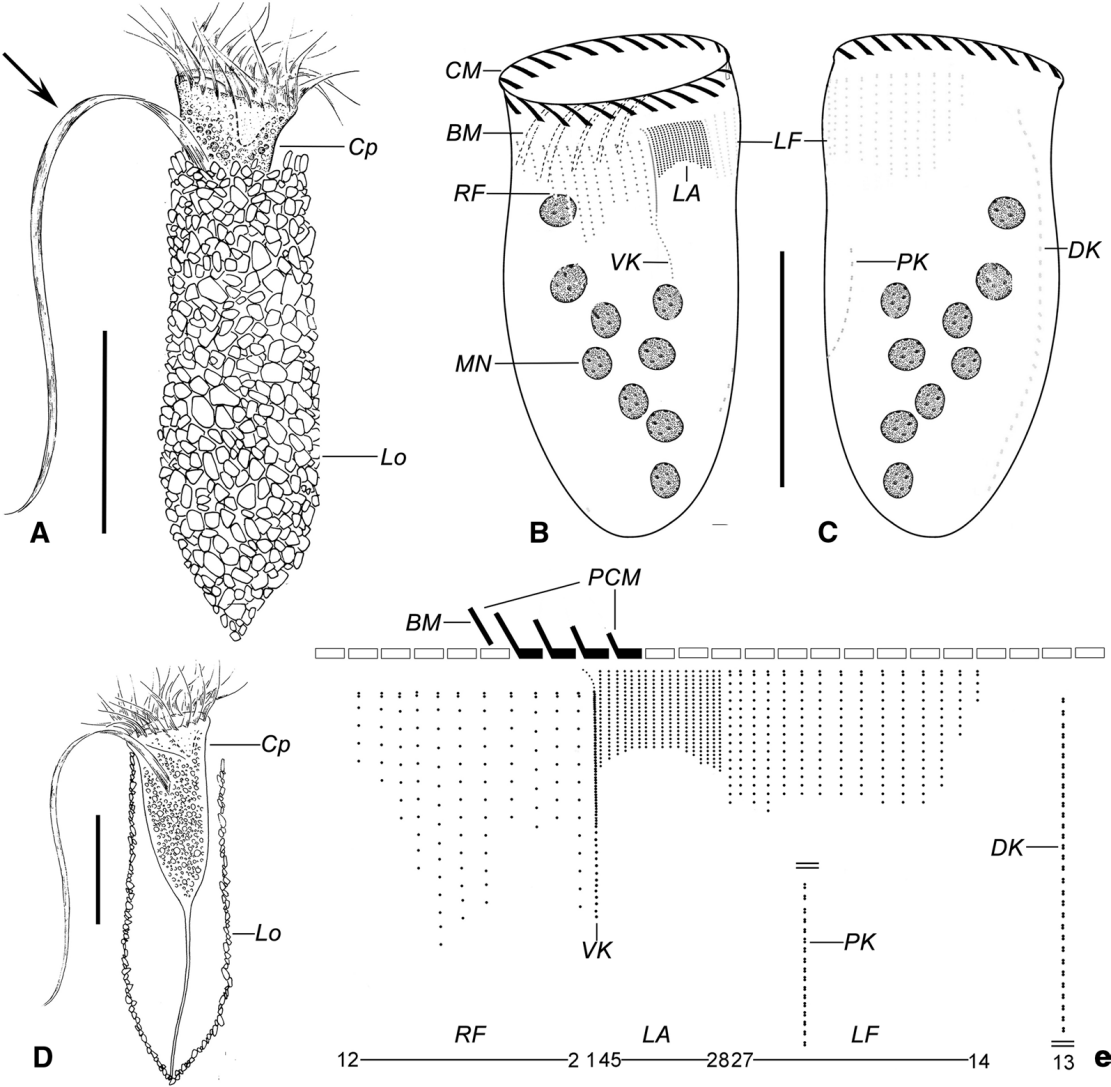
Drawing of *Antetintinnopsis gracilis* comb. nov. in vivo (**A**, **D**) and after protargol preparation (**B**, **C**, **E**). **A** Ventral view of a representative specimen, arrow shows the elongated ventral ciliary tuft. **B**, **C** Ciliary pattern of ventral (**B**) and dorsal (**C**) sides of the same specimen. **D** Ventral view of an extended specimen showing the details of cell proper. **E** Kinetal map of a morphostatic specimen. BM, buccal membranelles; CM, collar membranelles; Cp, cell proper; DK, dorsal kinety; LA, lateral ciliary field; LF, left ciliary field; Lo, Lorica; MN, Macronuclear nodules; PCM, prolonged collar membranelles; PK, posterior kinety; RF, right ciliary field; VK, ventral kinety. Scale bars = 40 μm (**A**, **B**), 30 μm (**C**)

**Fig. 5:**
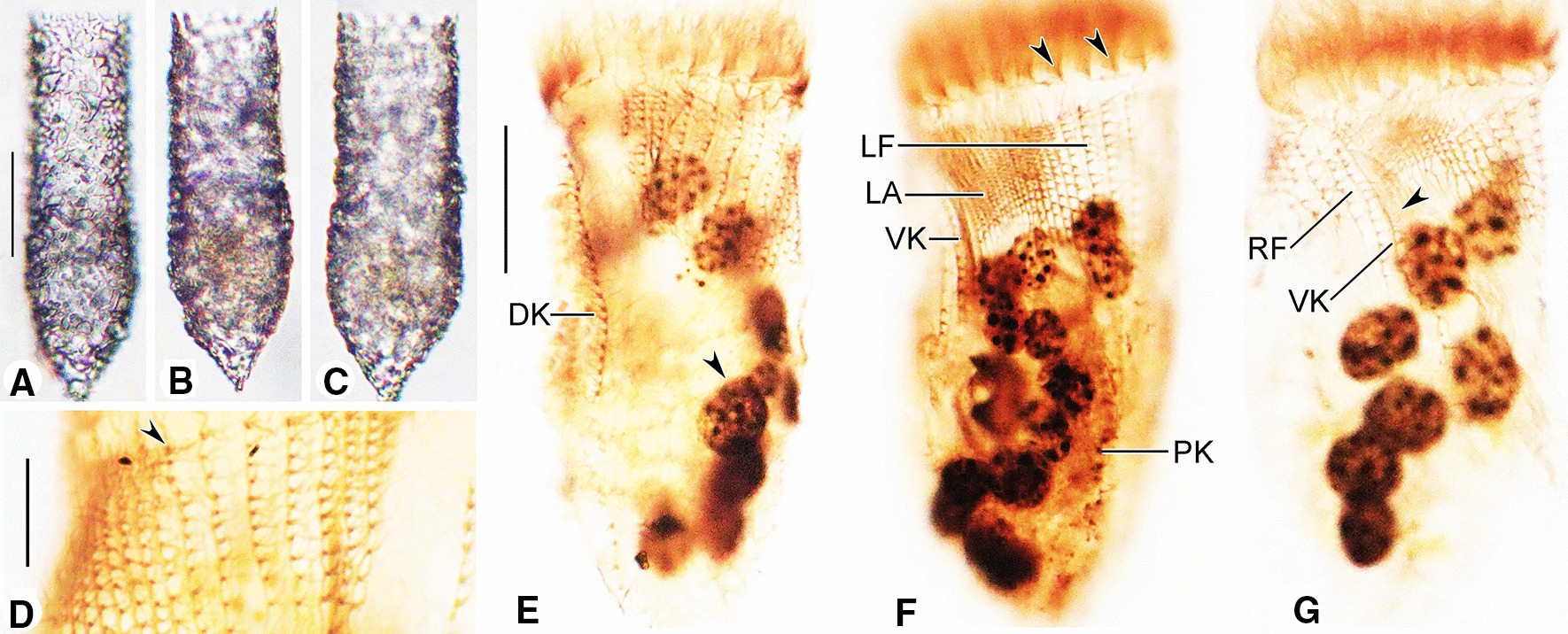
Photomicrographs of *Antetintinnopsis gracilis* comb. nov. in vivo (**A**–**C**) and after protargol staining (**D**–**G**). **A**–**C** Variations in lorica shape of Qingdao population. **D** Kinetidal distribution in left ciliary field, arrowhead marks the anteriormost dikinetids. **E**–**F** Detail views of the ciliary pattern in ventral (**F**) and dorsal (**E**) side, arrowhead in (**E**) indicates the macronucleus; arrowheads in (**F**) show collar membranelles. **G** The basal bodies of elongated ventral ciliary tuft (arrowhead). DK, dorsal kinety; LA, lateral ciliary field; LF, left ciliary field; PK, posterior kinety; RF, right ciliary field; VK, ventral kinety. Scale bars = 30 μm (**A**, **E**), 8 μm (**D**)

*Improved diagnosis based on original and Qingdao populations*. Lorica on average 105 μm long, with an opening usually 30–45 μm in diameter; bullet-shaped, composed of a cylindrical collar and an obconical bowl with pointed posterior end. Bowl on average 45 μm in width. Lorica opening usually narrower than bowl. Cell about 70–85 × 25–40 μm after protargol staining. On average ten ellipsoidal macronuclear nodules. Ventral kinety commencing anteriorly to first kinety of right ciliary field. Right ciliary field with on average 12 kineties. Dorsal kinety composed of on average 40 dikinetids. Left ciliary field and lateral ciliary field with on average 15 and 16 kineties, respectively. Posterior kinety composed of on average 14 dikinetids. About 23 collar membranelles, of which four extend into buccal cavity, one buccal membranelle.

*ZooBank registration number*. 9F26D6CB-DA3C-4A70-9E2D-C23509A255F3.

*Deposition of neotype and voucher specimens*. A protargol slide including the neotype specimen (Fig. 5E, G) was deposited in the Laboratory of Protozoology, Ocean University of China (registration number: WR2017101603-1). One additional protargol slide with voucher specimens was deposited in the same collection (registration number: WR2017101603-2).

*Description based on Qingdao population*. Lorica slender, composed of a cylindrical collar and an obconical bowl with a posterior angle of 60° (Figs. 4A, D, 5A–C); 95–115 μm long, opening of lorica about 35–44 μm in diameter, ratio of length to opening diameter about 3.3–2.8:1; bowl usually slightly wider than opening, about 39–55 μm wide. Wall densely agglutinated with irregular particles, and flake-like mineral, up to 7 μm across.

Cell obconical when fully extended, size about 75–90 × 22–35 μm in size in vivo and 70–85 × 26–39 μm after protargol staining (Figs. 4D, 5F, G). Posterior cell portion narrowed progressively forming a peduncle about 40 μm long that is attached to bottom of lorica (Fig. 4D). Ellipsoidal or irregular-shaped macronuclear nodules scattered in cytoplasm, seven to 12 in number, 9 µm in diameter/cross section each, and with several spherical nucleoli (Figs. 4B, C, 5E–G). Contractile vacuole, cytopyge, striae, accessory combs, tentaculoids, and capsules not recognizable. Cytoplasm colorless; food vacuoles about 3–6 μm across containing microalgae (e.g. diatoms). Locomotion usually by swimming while rotating about main cell axis, but rapidly reversing on contact with obstacles. When disturbed, cell retracts quickly into lorica.

Somatic ciliature composed a ventral, dorsal, and posterior kinety as well as a right, left, and lateral ciliary field (Figs. 4B, C, E, 5D–G). Kinetids of each ciliary row ostensibly connected by argyrophilic fibers (Fig. 5G). Ventral kinety commencing about 3 μm posteriorly to collar membranelles and 2 μm anteriorly to first kinety of right ciliary field, extending parallel to last kinety of left ciliary field with three portions: i. anterior portion that is about 5 μm long, usually composed of six monokinetids each of which bears a cilium that is 1 μm long cilium; kinetids spaced ca. 1 μm apart; ii. middle portion that is about 20 μm long, consisting of extremely densely arranged of kinetids (kinetids’ number not quantified) with long cilia that form the ciliary tuft; whose length of ciliary tuft could not be determined in vivo because it was always lying within the lorica, however, it appears to be at least as long as the cell length. iii. posterior portion which terminates in mid-region of cell; composed of about 15 widely spaced monokinetids spaced 1–2 μm apart, each bearing a 2 μm long cilium (Figs. 4B, E, 5G). Right ciliary field separated from collar membranelles by a 5 μm gap, composed of 10–13 kineties that are highly variable in length; neighboring kineties about 2–3 μm apart; each kinety consisting of about five to 15 monokinetids and one anterior dikinetid; cilia in right field about 2 μm long except for the anterior one in each dikinetid which measure about 7 μm after protargol staining (Figs. 4B, E, 5G). Dorsal kinety commencing about 9 μm posteriorly to collar membranelles, separated from right and left ciliary fields by conspicuously broad, unciliated stripes that are about 7 μm wide; curving slightly rightward curvature, and terminating posteriorly below rightmost kinety of right ciliary field; about 72 μm long and composed of 30–50 dikinetids, only posterior basal body of each dikinetid bears a cilium that is about 2 μm long after protargol staining (Figs. 4C, E, 5E). Left ciliary field commencing about 3 μm posteriorly to collar membranelles, consisting of 12–17 kineties; neighboring kineties 2–5 μm apart; all the kineties composed of one anterior dikinetid and 3–15 widely spaced monokinetids; each basal body bears a cilium about 3 μm long except for anterior basal body of each dikinetid, which has a cilium that is about 10 μm long after protargol staining (Figs. 4B, C, E, 5H). Lateral ciliary field located between ventral kinety and left ciliary field, commencing at same level as ventral kinety, composed of 14–20 monokinetidal kineties that slightly decrease in length from both sides towards middle; kinetids in each kinety densely spaced (less than 1 μm apart, numbers of kinetids not determined), cilia in lateral ciliary field about 2 μm long after protargol staining (Figs. 4B, E, 5F). Posterior kinety usually commencing about 10 μm posteriorly below left ciliary field and extending leftward curvature to the posterior portion of cell, about 25 μm long and consisting of on average 14 dikinetids, with only the posterior basal body bearing a cilium about 2 μm long after protargol staining (Figs. 4B, C, E, 5F).

Oral apparatus occupying anterior cell portion. Adoral zone of membranelles closed, orthogonal to main cell axis, consisting of 21–25 collar membranelles that are separated from each other by a 4 μm gap; invariably one buccal membranelle. Cilia of collar membranelles about 35 μm long. Polykinetids of four proximalmost collar membranelles progressively elongated (Figs. 4A, D, 5E, F). Endoral membrane and argyrophilic fibers associated with oral apparatus insufficiently impregnated by protargol to be observed.

### *Tintinnopsis tocantinensis* Kofoid & Campbell, 1929 (Fig. 6; Table 1)

**Fig. 6:**
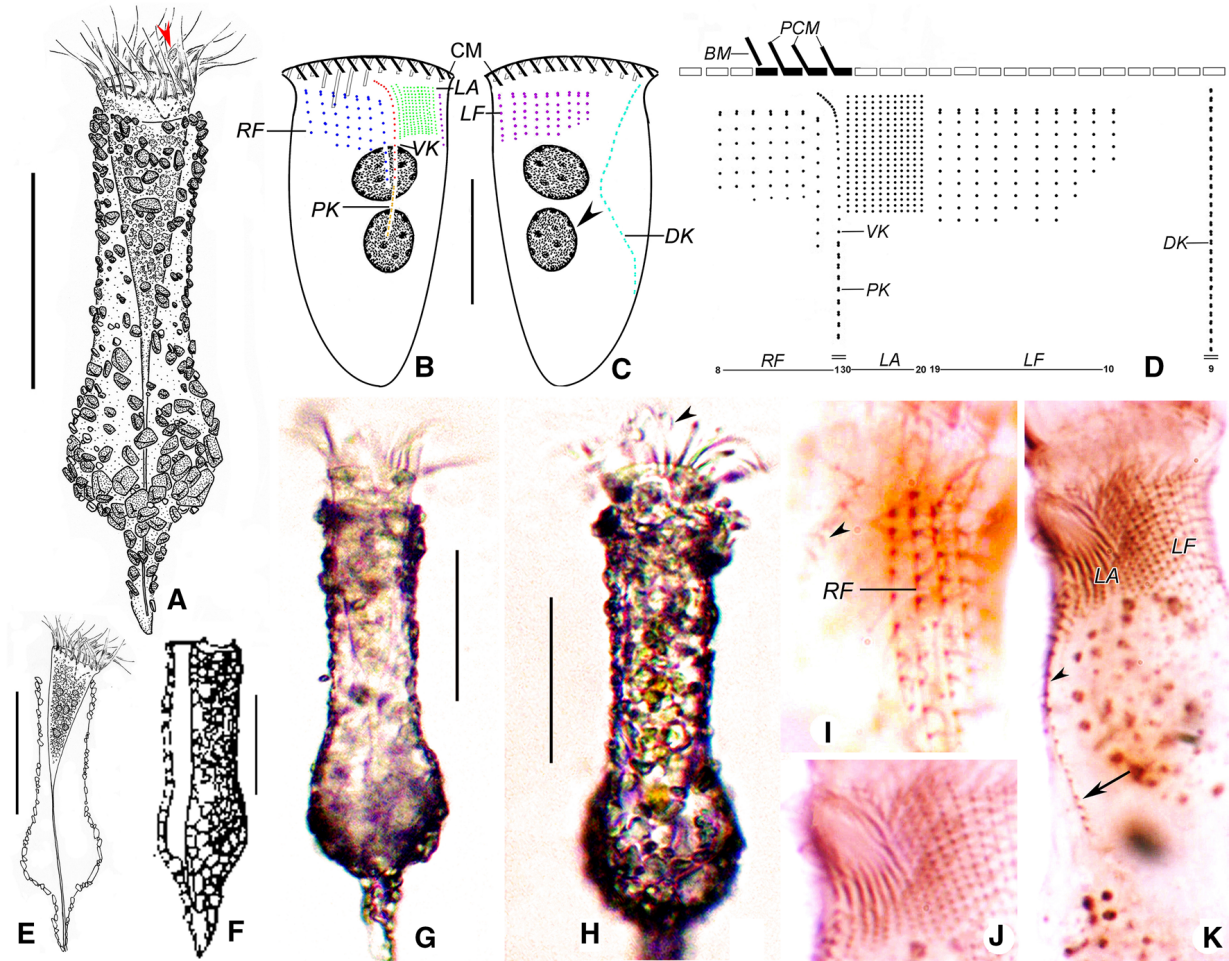
*Tintinnopsis tocantinensis* in vivo (**A**, **E**–**H**) and after protargol staining (**B**–**D**, **I**–**K**). **A** Ventral view of a representative individual. **B**, **C** Ciliary pattern of ventral and dorsal sides of the same specimen from Taizhou population, arrowhead indicates the macronucleus. **D** Kinetal map of a morphostatic specimen. **E** Ventral view of an extended cell in the lorica. F From Kofoid & Campbell [26]. **G** Ventral view of a living cell of *T. tocantinensis* from Haikou. **H** Details of the lorica wall, arrowhead indicates the tentaculoids. **I** Details of the ciliary pattern on dorsal side, arrowhead indicates DK. **J** Details of the ciliary pattern on ventral side. **K** Ciliary pattern on ventral side, arrowhead refers to ventral kinety and arrow shows the posterior kinety. BM, buccal membranelle; CM, collar membranelles; DK, dorsal kinety; LA, lateral ciliary field; LF, left ciliary field; PCM, prolonged collar membranelles; PK, posterior kinety; RF, right ciliary field; VK, ventral kinety. Scale bars = 60 μm (**A**, **E**–**H**), 30 μm (**B**–**C**)

*Remarks*. An improved diagnosis based on a Shenzhen population was provided by Jiang et al. [48]. The current population corresponds well with the Shenzhen specimens from the South China Sea. Hence, we only provided a redescription based on a new population from the East China Sea at Taizhou.

*Deposition of voucher slides*. Three voucher slides (registration numbers: WR2018061501-1, WR2018061501-2, and WR2018061501-3) with protargol stained specimens were deposited in the Laboratory of Protozoology, Ocean University of China.

### Description based on Taizhou population

Lorica 110–158 μm long, tripartite, i.e., composed of a cylindrical portion, a bulbous bowl, and a posterior projection. Lorica opening 15–35 μm in diameter. Cylindrical part as wide as aperture, 40–80 μm long. Bulbous part ovoid, on average 48 μm wide. Posterior portion tapered, stout, about 32 μm in length, usually open at rear end (Fig. 6A, E, G, H). Ratio of bowl length to lorica length about 0.28; ratio of posterior projection length to lorica length about 0.4. Lorica wall densely agglutinated with mineral particles (Fig. 6H).

Cell proper 45–80 × 17–33 μm in fully extended living specimens and 42–75 × 20–35 μm after protargol staining. Posterior end narrowed and always forming a peduncle (up to 55 μm long), which attaches to tapered portion of lorica (Fig. 6A, E, G, H). Invariably two globular macronuclear nodules in mid-region of cell, about 8–23 × 7–19 μm in size after protargol staining (Fig. 6B, C, K). Striae, accessory combs, contractile vacuole, cytopyge, and capsules not recognized in either living or stained specimens. Tentaculoids between membranelles recognizable in vivo, about 15 μm long (Fig. 6A, H). Cytoplasm colorless, containing food vacuoles up to 7 μm across with diatoms and microalgae (Fig. 6H). Locomotion by swimming while rotating about main cell axis, rapidly reversing on contact with obstacles. Cell retracts through quickly into lorica when disturbed; on cessation of disturbance, cell slowly extends through lorica aperture spreading its collar membranelles almost perpendicularly to main cell axis and resumes swimming and feeding.

Somatic ciliature consisting of a ventral, dorsal, and posterior kinety as well as a right, left, and lateral ciliary field (Fig. 6B–D, I–K). Ventral kinety commencing 4 μm posteriorly to collar membranelles, curving slightly leftwards before extending posteriorly in parallel to kineties of lateral ciliary field and terminating at mid-portion of cell proper; composed of densely spaced monokinetids in anterior portion but more widely ones in posterior portion; on average 28 μm long with about 28 monokinetids; cilia about 3 μm long after protargol staining (Fig. 6B, D, K). Right ciliary field composed of seven kineties, each separated from collar membranelles by a 7 μm gap except for first kinety which starts about 1 μm posteriorly to other kineties; each kinety consisting of six widely spaced monokinetids and one anterior dikinetid expect for the first kinety which has nine monokinetids; cilia of right field about 3–5 μm long except for elongated cilium of each dikinetid which is about 7–10 μm after protargol staining (Fig. 6B, D, I). Dorsal kinety dikinetidal, commencing about 5 μm posterior to collar membranelles, separated from right and left ciliary fields by conspicuously broad, unciliated stripes that are about 5 μm and 10 μm wide, respectively; anterior and posterior portions extending rightward and leftward, respectively; consisting of 31 dikinetids on average, posterior basal body of each kinetid bearing a cilium that is about 3 μm long after protargol staining (Fig. 6C, D, I). Left ciliary field separated from collar membranelles by a 6 μm unciliated stripe; consisting of nine kineties that slightly increase in length in clockwise direction (when viewed from apical aspect); each kinety consisting of monokinetids and one anterior dikinetid; cilia of left field about 2 μm long, except for elongated anterior cilia of dikinetids measuring about 6 μm after protargol staining (Fig. 6B–D, J, K). Lateral ciliary field with on average nine monokinetidal kineties, commencing about 5 μm posterior to collar membranelles except for first kinety which commences about 1 μm anteriorly to second kinety of right field; almost parallel to the ventral kinety; monokinetids more densely spaced in right portion than in left portion of field (Fig. 6B, D, J, K). Posterior kinety composed of 11 dikinetids; usually commencing below ventral kinety, about 2 μm posteriorly to posteriormost kinetosome of ventral kinety; 18 μm long on average; only posterior basal body of each dikinetid bears a cilium that is 2 μm long (Fig. 6B, D, K).

Adoral zone of membranelles closed, lying orthogonal to main cell axis; consists of 20–24 collar membranelles with cilia up to 25–30 μm long, including three that are significantly prolonged, polykinetids of which terminating 6–12 μm posteriorly to apical cell end. Single buccal membranelle (Fig. 6A–E). Endoral membrane insufficiently stained to be observed.

### Gene sequences and phylogenetic placement (Figs. 7, 8; Table 2; Additional file 4)

**Fig. 7:**
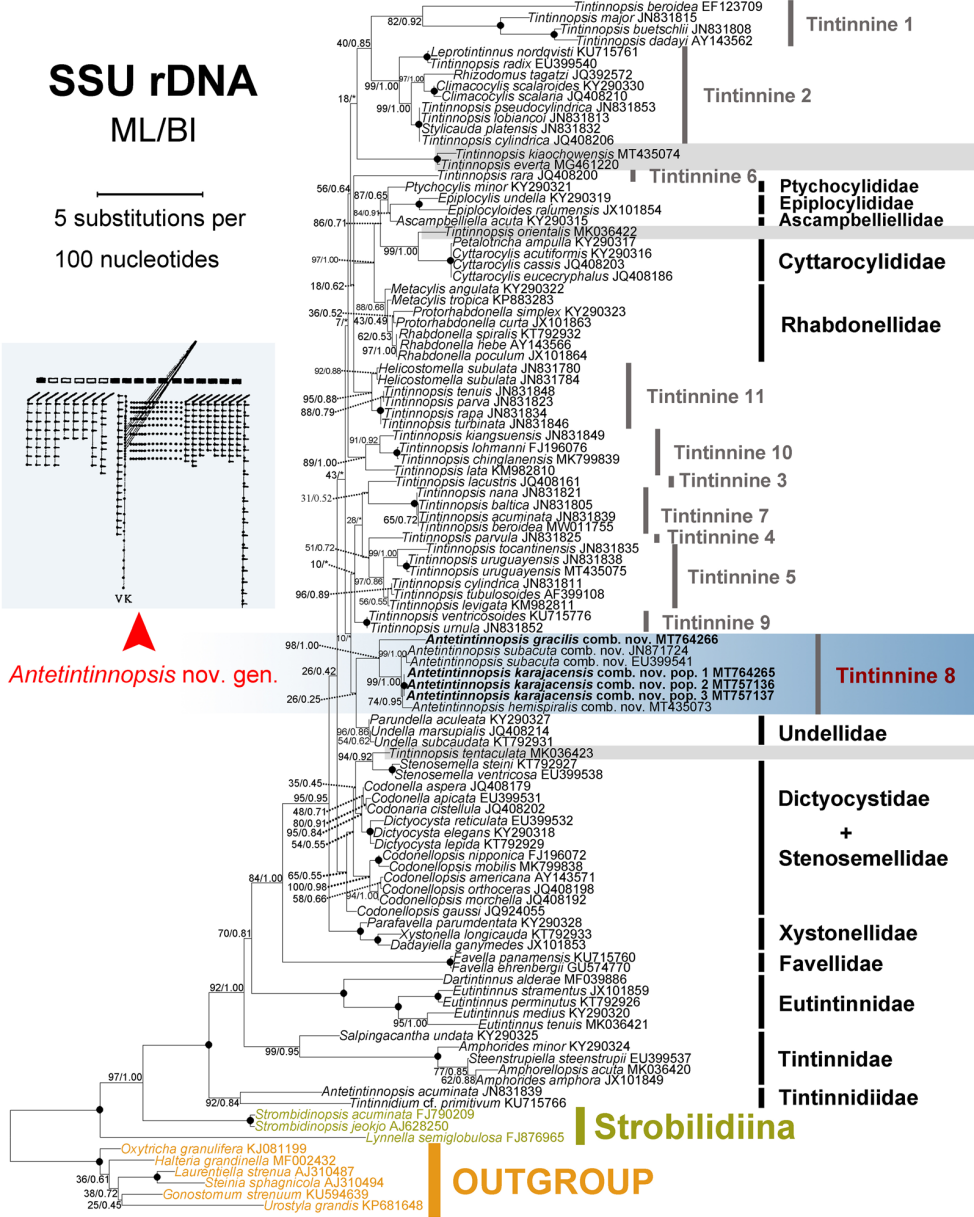
Phylogenetic tree inferred by maximum likelihood (ML) analyses of SSU rDNA sequences. Newly sequenced species are shown in bold font. Numbers at the nodes are the bootstrap values of the ML and posterior probabilities of the Bayesian inference (BI) analyses, respectively. Asterisks indicate discrepancies in the topologies of the ML and BI trees, thus only the values of ML are shown in these cases; black circles denote fully supported nodes. Numbering of tintinnid clades follows Santoferrara et al. [52], except for sequences marked in grey block. The scale bar corresponds to five substitutions per 100 nucleotide positions

**Fig. 8:**
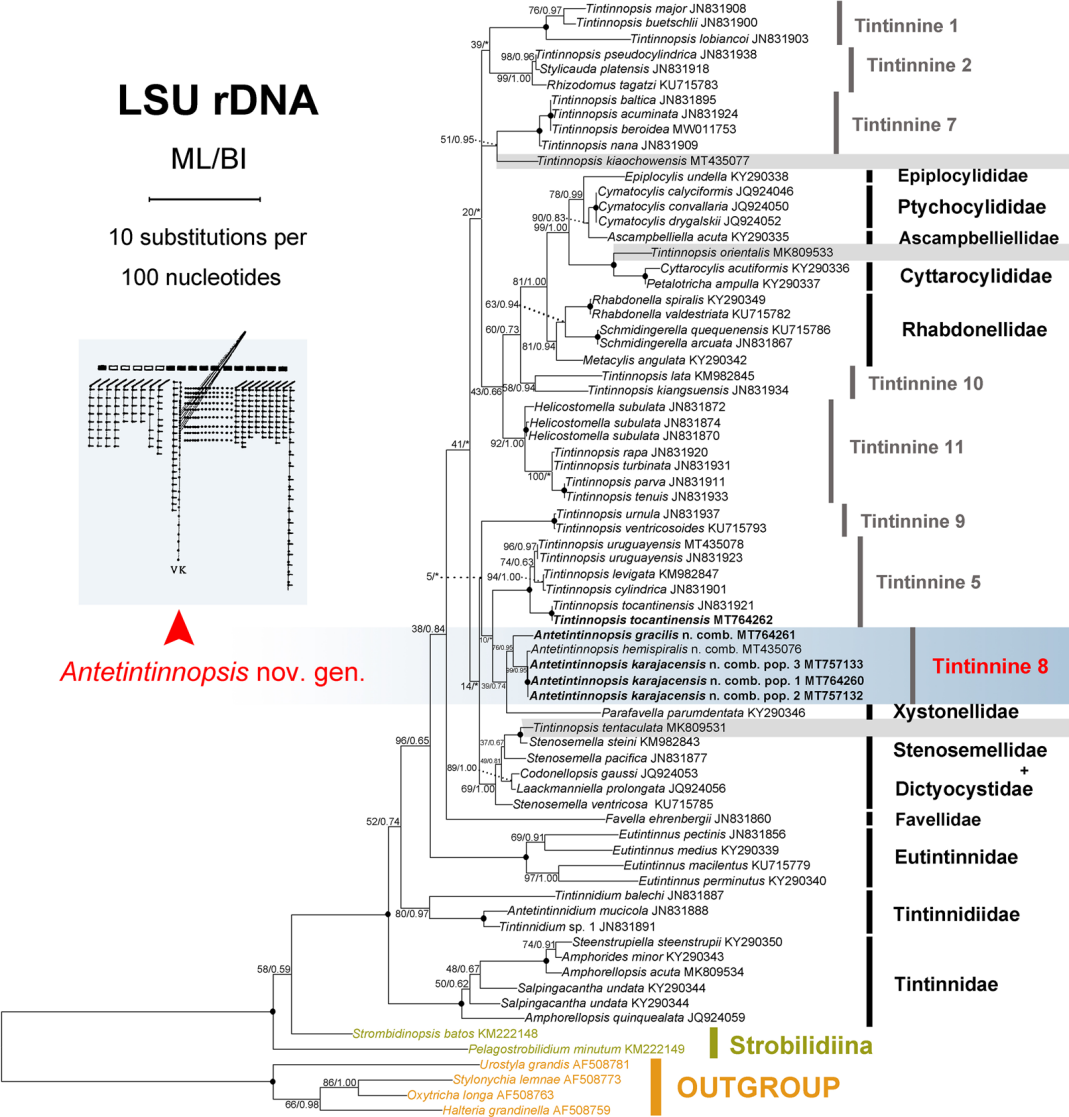
Maximum likelihood (ML) tree inferred from LSU rDNA sequences showing nodal support for ML and BI analyses. Newly sequenced species are shown in bold font. Asterisks indicate discrepancies in the topologies of the ML and BI trees, thus only the values of ML are shown in these cases; black circles denote fully supported nodes. Numbering of tintinnid clades follows Santoferrara et al. [52], except for sequences marked in grey block. The scale bar corresponds to ten substitutions per 100 nucleotide positions

**Table 2:** NCBI accession number, length, and GC content of SSU rDNA and LSU rDNA for species that are newly sequenced

Species	SSU rDNA sequences			LSU rDNA sequences		
	Acc. no	Length of seq	GC%	Acc. no	Length of seq	GC%
*Antetintinnopsis karajacensis* comb. nov. (pop. 1)	MT764265	1640 bp	47.56	MT764260	1745 bp	51.06
*Antetintinnopsis karajacensis* comb. nov. (pop. 2)	MT757136	1640 bp	47.56	MT757132	1745 bp	51.06
*Antetintinnopsis karajacensis* comb. nov. (pop. 3)	MT757137	1640 bp	47.56	MT757133	1745 bp	51.06
*Antetintinnopsis gracilis* comb. nov	MT764266	1633 bp	47.64	MT764261	1750 bp	51.37
*Tintinnopsis tocantinensis*	–	–	–	MT764262	1750 bp	50.57

All new sequences were deposited in the NCBI database, with the accession numbers, lengths, and GC contents of each as shown in Table 2. For both gene marker, the topologies of the BI and ML trees are basically congruent with variable support value, therefore only the ML tree topology (with nodal support from both methods) is shown for each (Figs. 7, 8). In the phylogenetic analysis of SSU rDNA sequence data, *Tintinnopsis*-like species clusters into 14 clades including 11 previously named clades. Our new SSU rDNA sequences of the three populations of *Antetintinnopsis karajacensis* comb. nov. are identical and differ from *A. gracilis* comb. nov. by 48 bp, from the Rhode River population of *A. subacuta* comb. nov. (JN871724) by 11 bp, from the Florida population of *A. subacuta* comb. nov. (EU399541) by 13 bp, from *A. hemispiralis* comb. nov. by 8 bp, and from *Tintinnopsis* spp. by 36–165 bp. Topologically, these four *Antetintinnopsis* species form a clade with strong support (98% ML, 1.00 BI; corresponding to clade 8 in Fig. 7). In this clade, *A. karajacensis* comb. nov. first clusters with *A. hemispiralis* comb. nov. (MT435073) with moderate to high support (74% ML, 0.95 BI) and then with *A. subacuta* comb. nov. with strong support (99% ML, 1.00 BI). *Antetintinnopsis gracilis* comb. nov. occupies the basal position in this clade. Additionally, the Haikou population of *Tintinnopsis tentaculata* (MK036423) was found to be most closely to two species of *Stenosemella* (94%ML/0.92BI); *Tintinnopsis orientalis* (MK036422) is sister to the clade formed by species of Cyttarocylididae with maximum support. *Tintinnopsis everta* (MG461220) and *T. kiaochaowensis* (MT435074) group together with full support.

In terms of the LSU rDNA sequences, *Antetintinnopsis karajacensis* comb. nov. differs from *Antetintinnopsis gracilis* comb. nov. by 33 bp, from *Antetintinnopsis hemispiralis* comb. nov. (MT435076) by 78 bp, and from *Tintinnopsis* spp. by 81–210 bp. In phylogenetic trees based on analyses of LSU rDNA sequence data, eight clades for *Tintinnopsis*-like species, including the *Antetintinnopsis* gen. nov. cluster (clade 8), were recovered (Fig. 8), which basically corresponds with the SSU rDNA analyses. Furthermore, the evolutionary relationships within the *Antetintinnopsis* gen. nov. clade are also consistent with those in the SSU rDNA tree. The newly sequenced Taizhou population of *T. tocantinensis* (MT764262) clusters with the Long Island population (JN831921) with full support forming a group that is sister to the *Tintinnopsis cylindrica* (JN831901) + *T. levigata* (KM982847) + *T. uruguayensis* (JN831923 and MT435078) clade with maximum support.

## Discussion

### Establishment of the new genus *Antetintinnopsis* gen. nov.

Historically, lorica features have been the main diagnostic character used to delineate genera in Tintinnina owing to their ease of collection and observation. In recent decades, however, the application both of cytological characters based on live and stained specimens and phylogenetic analyses based on sequence data has led to the traditional lorica-based classification being challenged [e.g., 29, 30, 53]. Hence, detailed investigations of cytological features of cell are necessary in order to distinguish among forms with a similar lorica. In the present study we revealed that the complex ciliary pattern in *T. karajacensis* and *T. gracilis*, is sufficiently distinct to distinguish these from most other tintinnines as follows: i. kinetids in the ventral kinety conspicuously densely arranged in the middle portion as compared with anterior and posterior portions (vs. basically even distributed); ii. kinetids in the middle portion of the ventral kinety bear extremely long cilia that form a ciliary tuft (vs. absent) [e.g., 47–54]. However, two species of *Tintinnopsis* (*T. subacuta* and *T. hemispiralis*) cannot be separated from *T. karajacensis* and *T. gracilis* based on these characters. *Tintinnopsis subacuta*, for example, was described as having both of these two distinctive features [49, 55]. Most recently, the elongated ciliary tuft was also recognized in *T. hemispiralis* [45]. Consequently, we here establish *Antetintinnopsis* gen. nov. for the four species of *Tintinnopsis* with these two synapomorphies, and assign them to the new genus as follows: *Antetintinnopsis gracilis* (Kofoid & Campbell, 1929) comb. nov., *Antetintinnopsis hemispiralis* (Yin, 1956) comb. nov., *Antetintinnopsis karajacensis* (Brandt, 1896) comb. nov., and *Antetintinnopsis subacuta* (Jörgensen, 1899) comb. nov. Furthermore, phylogenetic analyses based on sequence data from two nuclear ribosomal loci support the validity of the new genus.

## Type designation within the new genus

*Antetintinnopsis hemispiralis* (Yin, 1956) comb. nov. is selected as type species because its description is based on not only silver staining but also live observations, and type materials are available following the designation of a neotype specimen by Bai et al. [45]. Furthermore, its ribosomal gene sequence data were first available for a member of this genus [45].

### Comments on *Antetintinnopsis karajacensis* (Brandt, 1896) comb. nov.

An adequate sample size avoids the establishment of new combination based on possibly atypical specimens and allows rough estimates of the intraspecific variability [35]. In this sense, it is also important to study several populations wherever possible. In the present study, specimens with congruent lorica morphologies and cell features were collected in Haikou and Beihai which are within a comparatively short distance of each other (no more than 200 km apart) along the southern Chinese coast of the South China Sea. However, specimens of a third population collected from Zhoushan on the East China Sea coast, about 1900 km away from Haikou, show smaller dimensions of both lorica and cell when compared with the two other populations. Nevertheless, all the specimens agree well in terms of the diameter of the lorica opening and the presence of an elongated ciliary tuft, plus they have identical SSU rDNA sequences. Hence, we believe that the size differences are very likely environment- and/or population- dependent.

*Antetintinnopsis karajacensis* comb. nov. was originally isolated by Brandt from Karajak-Fjord in Davis Strait and was described under the name *Tintinnopsis karajacensis* based exclusively on lorica features (Fig. 1D) [57]. We identified our three populations as being conspecific with this taxon because all of them correspond well with the type population in the following features: i. lorica structure (hard and entirely agglomerated) and shape (cylindrical and rounded at the aboral end); ii. opening diameter of lorica (Zhoushan population: 35–40 μm, Beihai population: 35–40 μm, Haikou population: 40–45 μm, type population: about 40 μm).

To date, more than ten populations have been described worldwide under the name *Tintinnopsis karajacensis* by having a cylindrical agglutinated lorica, which is a dominant form shared by different tintinnine species in the pelagial of marine and brackish coastal waters. Moreover, they also present a considerable variability in lorica size [38]. Accordingly, based on these specimens there must be some misidentifications in the literature. Considering that the diameter and characteristics of the lorica opening are widely accepted as taxonomic characters, we recognize only eight of these populations of *Tintinnopsis karajacensis* as being congruent with the original population (Additional files 2, 5) [22, 27, 56–61].

Currently, two species of *Tintinnopsis* should be compared with our isolates in terms of their similarity in overall lorica shape, viz. *Tintinnopsis beroidea* Stein, 1867, and *Tintinnopsis rotundata* Kofoid & Campbell, 1929. *Tintinnopsis beroidea* differs from our specimens in having a slender aboral portion, more oral membranelles (~ 30 vs. 23), and fewer macronuclear nodules (2 vs. ~ 5) [26, 36]. *Tintinnopsis rotundata* differs from our specimens in having a rounded posterior end and a ragged lorica opening rim [26].

Regarding cell features, *Antetintinnopsis hemispiralis* (Yin, 1956) comb. nov., *A. subacuta* (Jörgensen, 1899) comb. nov. and *A. gracilis* (Kofoid & Campbell, 1929) comb. nov. resemble *A. karajacensis* comb. nov. not only in the complex ciliary pattern, but also in the presence of multiple macronuclear nodules [45, 49, 55]. However, both *A. hemispiralis* comb. nov. and *A. gracilis* comb. nov. can be separated from *A. karajacensis* comb. nov. by having an obconical (vs. slightly rounded) aboral end and by the number of macronuclear nodules (7–11, on average 9; 7–12, on average 10 vs. 2–8, on average 4 or 5 in *A. karajacensis* comb. nov.) [45]. *Antetintinnopsis subacuta* comb. nov. can be separated from *A. karajacensis* comb. nov. by its vase-like lorica with a subspherical bowl that is wider than the lorica opening (vs. subcylindrical with slightly rounded aboral end in *A. karajacensis* comb. nov.) [49, 55]. Furthermore, differences in their ribosomal gene sequences also support the distinction between these forms at species level [51].

### Comments on *Antetintinnopsis gracilis* (Kofoid & Campbell, 1929) comb. nov.

*Tintinnopsis gracilis* was established by Kofoid & Campbell [26] to describe a morphotype previously recorded by Brandt [62] as *Tintinnopsis karajacensis* var. α from Schott off the West Coast of Borneo. Since the cell features of the Schott population are unknown, species identification is only based on lorica morphology. The Qingdao specimens match the original illustrations of Brandt (1907) in terms of lorica dimensions (length: 95–115 μm vs. 110–135 μm in original population; opening diameter: 31–40 μm vs. 30–41 μm in original population) and shape (both are elongate with aboral end subconical and lack spiral turns) [62]. Hence, the Qingdao population is considered to be conspecific with *T. gracilis*.

Populations worldwide have been continuously reported under the name *T. gracilis*, almost all of which correspond well with the original population in terms of morphometric data of the lorica [22, 26, 60, 63–67]. The lorica of Qingdao population matches the previous populations in terms of both the lorica dimension (lorica length 95–135 μm; lorica width 25–41 μm; opening diameter 28–39 μm) and the angle of the bowl’s posterior portion (40–75°) (Additional files 3, 6) [22, 26, 60, 63–67]. However, for all previously reported populations, the descriptions are cursory and data both for cell features and gene sequences are unavailable. Therefore, a detailed comparison with conspecific populations is impossible.

*Antetintinnopsis hemispiralis* (Yin, 1956) comb. nov., *A. subacuta* (Jörgensen, 1899) comb. nov., and *Tintinnopsis tubulosoides* resemble our specimens of *A. gracilis* comb. nov. in terms of the overall shape of the lorica and/or cell features. However, *A. subacuta* comb. nov. differs from *A. gracilis* comb. nov. by having a subspherical aboral end (vs. obconical aboral end) and fewer kineties in the left ciliary field (on average 11 vs. 14) [49, 55]. *Antetintinnopsis hemispiralis* comb. nov. can be separated from our specimens by the presence of 3–5 spiral striations in the collar of the lorica (vs. spiral striations absent), the commencing position of the ventral kinety (i.e., anteriorly to the second kinety vs. first kinety) of right ciliary field, and the length of the ventral kinety (extending to posterior one-quarter to one-third of cell proper vs. terminating at the middle portion of cell proper in *A. gracilis* comb. nov.) [45]. Furthermore, the SSU rDNA sequences of *A. subacuta* comb. nov. (EU399541) and *A. hemispiralis* comb. nov. (MT435073) has a dissimilarity of 2.8% (46 bp) and 2.9% (48 bp), respectively, compared with our new sequence of *A. gracilis* comb. nov., which represents an interspecies-level divergence in tintinnine ciliates [51]. *Tintinnopsis tubulosoides* can be separated from Qingdao population of *A. gracilis* comb. nov. by the presence (vs. absence) of spiral striations in the collar portion of the lorica [26].

### Comments on *Tintinnopsis tocantinensis* Kofoid & Campbell, 1929

*Tintinnopsis tocantinensis* can be recognized by its unique lorica appearance, i.e., lorica tripartite, composed of a cylindrical portion, a bulbous part, and a tapered portion. It was first established by Kofoid & Campbell [26] and reported described by Brandt [57] as *T. aperta* var. α (Fig. 6F). We identified Taizhou population as *T. tocantinensis* because it matches the original population very well in the lorica shape, although it is considerably longer (110–160 μm vs. 85 μm). Considering the variability of the lorica length in tintinnines [35], we believe that this variation is a population-dependent difference.

The infraciliature of *T. tocantinensis* has been previously reported on a population from Shenzhen, China [48]. Our Taizhou population resembles the Shenzhen population in both lorica features and ciliary pattern, which supports the conspecificity of both forms [48].

In terms of lorica shape, only *Tintinnopsis aperta* Brandt, 1906 resembles *T. tocantinensis* in having a cylindrical portion, a bulbous part, and a tapered portion. However, the latter can be distinguished from the former by its shorter (16–42 μm vs. 45–105 μm) and stouter tapered portion [57].

### Neotypification

Considering the requirement of Article 75.3.6 of the International Code of Zoological Nomenclature [68], we neotypify *A. karajacensis* comb. nov. and *A. gracilis* comb. nov. with two Chinese populations for the following reasons: i. no type specimens are available for either species; ii. the existing descriptions are too incomplete, e.g., they lack detail cytological and molecular information, to allow accurate identification; iii. neotype slides are of a good quality allowing the specific features to be clearly recognized. Unfortunately, both neotypes do not come from near the original type locality (Chinese coast water vs. Karajak-Fjord in Davis Strait, western Greenland and Schott off the west coast of Borneo, respectively). However, according to the rather wide distribution of both species, it seems justified to designate neotypes from different sites, especially as both sites are marine habitats that are connected by oceanic currents. Thus, this point should not be overinterpreted (for a thorough discussion of this problem, see Foissner et al. [69]).

### Phylogenetic analyses

Currently, phylogenetic relationships within Tintinnina are uncertain and the systematic positions of most *Tintinnopsis* species remain unresolved because of limited data [e.g., 29, 52]. Nevertheless, members of *Tintinnopsis* and related species were generally well grouped in the SSU rDNA and LSU rDNA trees, which corresponds with previous phylogenetic studies [52]. One well-supported monophyletic lineage should be concerned, which is marked as tintinnine 8 in Figs. 7 and 8, and consists of all *Antetintinnopsis* species, viz. *A. gracilis* comb. nov., *A. hemispiralis* comb. nov., *A. karajacensis* comb. nov., and *A. subacuta* comb. nov. These four species share some morphological synapomorphies, i.e., configuration of the ventral kinety, presence of an elongated ventral ciliary tuft, and multiple macronuclear nodules. These data provided strong evidence that the genus *Tintinnopsis* should be revised pending the congruence between morphological and molecular data, however, this taxonomic action should be carried out after most *Tintinnopsis*-like species are cytologically and genetically studied.

## Conclusions

The findings of the integrative morphological and phylogeny investigations on two poorly known *Tintinnopsis* species support the establishment of *Antetintinnopsis* gen. nov., and the assignment of two additional *Tintinnopsis* species to the new genus, based on their distinct somatic ciliary pattern and their clustering patterns in gene trees. Moreover, the results of our phylogenetic study based on sequences of two nuclear ribosomal loci provides compelling evidence that *Tintinnopsis* comprises several distinct evolutionary lineages, necessitating a detailed morphological review of these organisms. The taxonomic novelties reported in this study highlight the importance of integrative studies, that is, the combination of morphological and molecular characters of various populations, in resolving the systematics of tintinnines.

## Methods

### Sample collection and environmental factors (Fig. 9; Additional file 1)

**Fig. 9:**
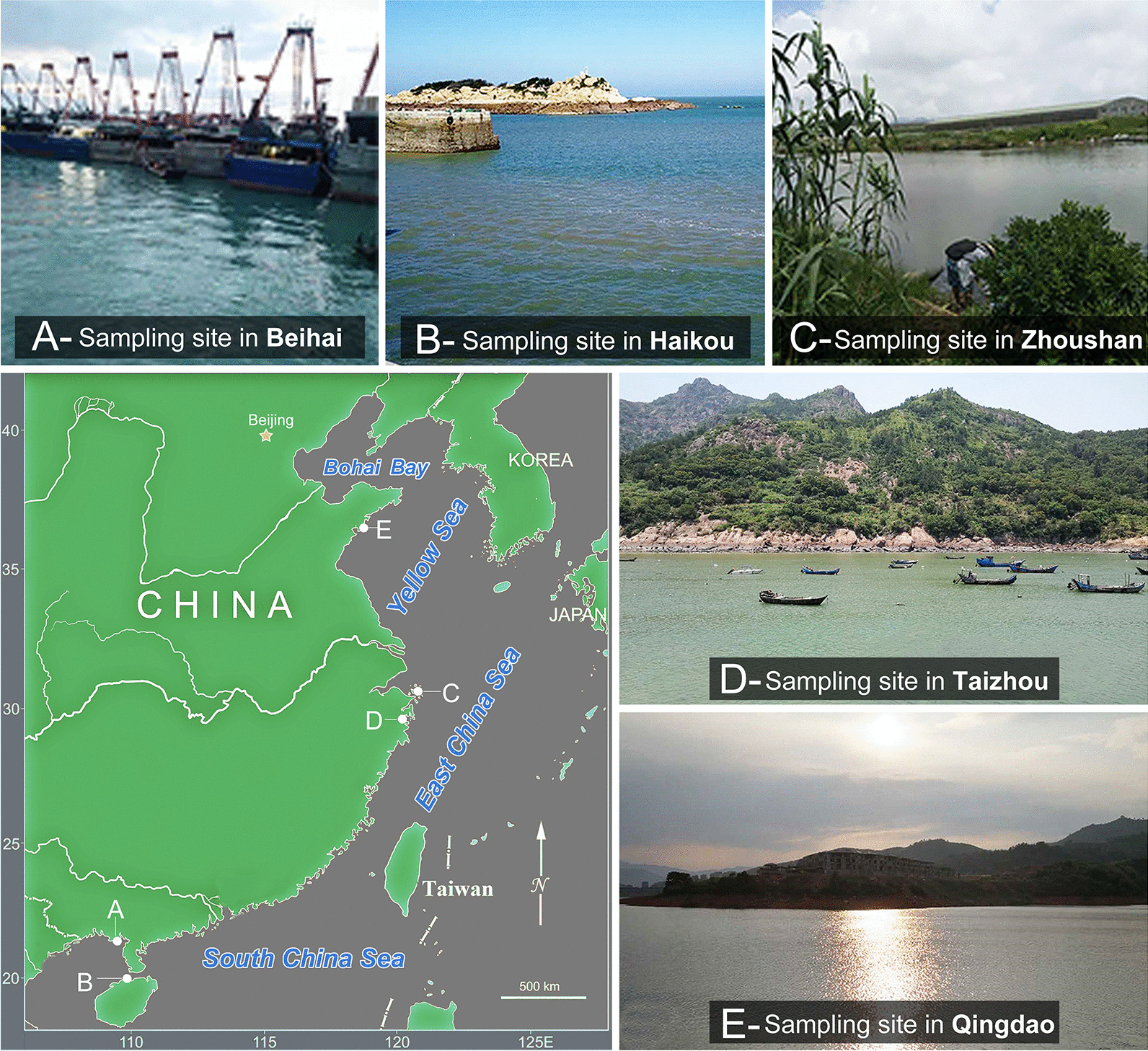
Locations and photographs of the sampling sites

All the tintinnine ciliates redescribed herein were collected from surface water (0–2 m depth) by horizontal towing of a 25-μm meshed plankton net at different sites on the coast of China. The Haikou population of *Antetintinnopsis karajacensis* comb. nov. was isolated from coastal waters of the South China Sea at Haikou Bay, Hainan Province (20°02′32″N, 110°17′2″E) in November 2017. The Zhoushan population of *A. karajacensis* comb. nov. was collected from coastal waters of the East China Sea at an inlet in Zhoushan, Zhejiang Province (29°57′2"N, 122°22′29"E) in October 2018. The Beihai population of *A. karajacensis* comb. nov. was collected from a port in the Beibu Gulf, Guangxi Province (21°24′29"N, 109°9′31"E) in July 2018. *Antetintinnopsis gracilis* comb. nov. was collected on 16 October 2017 from coastal waters of the Yellow Sea at a wharf in Qingdao, Shandong Province (35°44′31″N, 120°00′52″E). *Tintinnopsis tocantinensis* was isolated on 15 June 2018 from coastal waters off the East China Sea in Taizhou, Zhejiang Province (29°5′59″N, 121°39′27″E). Water temperature, salinity, and pH were measured in situ using a water quality-measuring instrument (YSI Professional Plus, America).

### Taxonomy studies

The samples were transferred into Petri dishes (5 cm across) and the ciliates were immediately isolated under a dissecting microscope (Guiguang XTL-200, China) for further study. Attempts to establish pure cultures were unsuccessful. Swimming motion and cell flexibility and contractility were observed in undisturbed specimens under a dissecting microscope. The morphology of living cells and lorica were investigated using bright-field and differential interference contrast microscopy (Olympus BX 51, Tokyo, Japan) at magnifications of 100–1000 × . Protargol staining was applied to reveal the infraciliature and nuclear apparatus [70]. The protargol reagent was made according to Pan et al. [71]. The cells were stained after removal of their lorica. Drawings of living cells were based on photomicrographs and those protargol-stained cells were made with the help of a camera lucida. In vivo measurements were performed at magnifications of 40–1000 × . Counts and measurements of stained specimens were performed at 400–1000 × magnifications. Terminology is according to Agatha & Riedel-Lorjé [39].

### DNA extraction, amplification and sequencing

Although clonal cultures were not established, we are confident that all morphological and molecular studies of each isolate dealt with a single species because i) all the five populations investigated here can be easily recognized by their lorica features and no cryptic species were present in the subsamples, and ii) no other tintinnine morphotypes were present in the protargol preparations. For each population, a single specimen was observed in detail at high magnification (up to 1000 ×) by careful evaluation of morphological features of the lorica and the cell, washed four or five times using sterilized, filtered in situ water to exclude potential contamination, and then transferred to a 1.5 mL microfuge tube with a minimum volume of water. The genomic DNA was extracted using the DNeasy Blood & Tissue kit (Qiagen CA) following the manufacturer’s instructions. The amplification of the SSU rDNA sequences was performed with the primers 18 s-F (5′-AAC CTG GTT GAT CCT GCC AGT-3′) and 18 s-R (5′-TGA TCC TTC TGC AGG TTC ACC TAC-3′) [72]. Amplification of the LSU rDNA sequences was performed with the primers F3 (5ʹ-ACC/C CGC TGA/G AT/CT TAA GCA T-3ʹ) and R2 (5ʹ-AAC CTT GGA GAC CTG AT-3ʹ) [73]. PCR amplifications were performed according to the following protocol: 98 °C for 30 s, followed by 18 cycles of 98 °C for 10 s, 69 °C for 40 s with the remaining cycles stepping down by 1 °C for each cycle; then 72 °C for 90 s and 18 cycles of 98 °C for 10 s, 51 °C for 40 s, 72 °C for 90 s; and a final extension at 72 °C for 4 min. Q5® Hot Start High-Fidelity DNA Polymerase (New England BioLabs, USA) was used to minimize the possibility of PCR amplification errors. The PCR products were sequenced bidirectionally by the Tsingke Biological Technology Company (Beijing, China).

### Molecular phylogeny

In addition to the nine newly obtained sequences, other sequences used in the present analyses were downloaded from the NCBI database, including six SSU rDNA and four LSU rDNA sequences of hypotrichs and *Halteria grandinella* as the outgroup taxa, respectively.

All sequences were aligned using the MUSCLE program package on the European Bioinformatics Institute web server). The resulting alignments were then edited manually with trimming both ends included 1705 sites of SSU rDNA (107 taxa) and 1292 sites of LSU rDNA (73 taxa). ML analyses of both SSU rDNA and LSU rDNA sequences were performed on the CIPRES Science Gateway (URL: http://www.phylo.org/sub_sections/portal) [74], with RAxML-HPC2 on XSEDE using the GTRGAMMA + I model as selected by Modeltest v. 3.4 [75]. Searches for the best tree were conducted starting from 100 random trees, and 1000 nonparametric bootstrap replicates were done to assess the reliability of the internal branches. BI analyses were performed on the CIPRES Science Gateway using MrBayes v. 3.1.2 on XSEDE with the GTR + I + Γ model selected by MrModeltest v. 2.2 (for details, see Additional file 7) [76]. Markov chain Monte Carlo (MCMC) simulations were run for a million generations with a sample frequency of every 100th generation. The first 25% of trees were discarded as burn-in. The number of chains to run was four. MEGA v7 was used to visualize the tree topologies [77].

Classification and naming of phylogenetic clades mainly follow Adl et al. [78] and Santoferrara et al. [52], respectively.

## Supplementary Information

**Additional file 1: Table S1.** Environmental factors of the sampling sites.

**Additional file 2: Table S2.** Morphometric data of *Antetintinnopsis karajacensis* comb. nov. from the literature matching our specimens in lorica shape.

**Additional file 3: Table S3.** Morphology data of *Antetintinnopsis gracilis* comb. nov. from the literature matching our specimens in lorica shape.

**Additional file 4: Fig. S1.** Percent identity matrix of *Antetintinnopsis karajacensis* comb. nov. and *A. gracilis* comb. nov. with other *Tintinnopsis*-like species based on SSU and LSU DNA sequences.

**Additional file 5: Fig. S2.** Geographical distribution and morphological comparisons of different populations of *Antetintinnopsis karajacensis* comb. nov. based on the literatures and the present study. The vector maps were originally downloaded from the open-access website at http://mapsopensource.com/. All line drawings from open-access articles or books distributed under the terms of the Creative Commons Attribution License. LL, lorica length; LOD, lorica opening diameter.

**Additional file 6: Fig. S3.** Geographical distribution and morphological comparisons of different populations of *Antetintinnopsis gracilis* comb. nov. based on the literatures and the present study. The vector maps were originally downloaded from the open-access website at http://mapsopensource.com/. All line drawings from open-access articles or books distributed under the terms of the Creative Commons Attribution License. LL, lorica length; LOD, lorica opening diameter.

**Additional file 7: Table S4.** Characterization of the datasets and evolutionary models used for Bayesian analyses.

## Data Availability

Sequences data are available in the NCBI database (Accession Numbers: MT764260, MT764261, MT764262, MT764265, MT764266, MT757123, MT757133, MT757136, and MT757137). Permanent slide containing the protargol-impregnated specimens were deposited in the Laboratory of Protozoology, Ocean University of China and the Laboratory of Protozoology, South China Normal University (Accession Codes: WR2017110301-1, WR2017110301-2, HT2018071924a, HT2018071924b, HT2018081435a, HT2018081435b, WR2017101603-1, WR2017101603-2, WR2018061501-1, WR2018061501-2, and WR2018061501-3). The datasets used and/or analyzed during the current study are available from the corresponding author on reasonable request.

## Abbreviations

BM: Buccal membranelle
CM: Collar membranelle
CV: Coefficient of variation in %
DK: Dorsal kinety
EM: Endoral membrane
FB: Fibers bundles
GTR + I + Γ: General time reversible + invariable sites + gamma model of nucleotide substitution
LA: Lateral ciliary field
LL: Lorica length
LOD: Lorica opening diameter
LF: Left ciliary field
LSU rDNA: Large subunit ribosomal DNA
M: Median
Ma: Macronuclear nodules
Max: Maximum
Mean: Arithmetic mean
Min: Minimum
*n*: Number of specimens examined
PCM: Prolonged collar membranelles
PK: Posterior kinety
pH: Potential of hydrogen
RF: Right ciliary field
S: Salinity
SD: Standard deviation
SSU rDNA: Small subunit ribosomal DNA
T: Water temperature. VK: ventral kinety
