# Correction to: Integrative taxonomy and molecular phylogeny of three poorly known tintinnine ciliates, with the establishment of a new genus (Protista; Ciliophora; Oligotrichea)

**DOI:** 10.1186/s12862-021-01875-w

**Published:** 2021-08-11

**Authors:** Rui Wang, Yang Bai, Tao Hu, Dapeng Xu, Toshikazu Suzuki, Xiaozhong Hu

**Affiliations:** 1 College of Fisheries, & Key Laboratory of Mariculture, Ministry of Education, Ocean University of China, Qingdao, 266003 China; 2 Institute of Evolution & Marine Biodiversity, Ocean University of China, Qingdao, 266003 China; 3 Laboratory of Protozoology, Key Laboratory of Ecology and Environmental Science in Guangdong Higher Education, South China Normal University, Guangzhou, 510631 China; 4 State Key Laboratory of Marine Environmental Science, Institute of Marine Microbes and Ecospheres, College of Ocean and Earth Sciences, Xiamen University, Xiamen, 361102 China; 5 Faculty of Fisheries, Nagasaki University, 1‑14 Bunkyo-machi, Nagasaki, 852‑8521 Japan

## Correction to: BMC Ecol Evol (2021) 21:115 https://doi.org/10.1186/s12862-021-01831-8

Following the publication of the original article [1], the authors have flagged that on page 2, “Antetintinnopsis hemispiralis” was mistakenly replaced by “Antetintinnopsis karajacensis” as type species.

The original article has been corrected.
