# Supplementary material for: Integrative taxonomy and molecular phylogeny of three poorly known tintinnine ciliates, with the establishment of a new genus (Protista; Ciliophora; Oligotrichea)
Source: BMC Ecol Evol. 2021 Jun 9;21:115. doi: 10.1186/s12862-021-01831-8 (PMC8243829; doi:10.1186/s12862-021-01831-8)
Supplement: Supplementary file 1 — Additional file 1: Table S1. Environmental factors of the sampling sites. [file 12862_2021_1831_MOESM1_ESM.docx]

| **Site** | **T (℃)** | **S (ppt)** | **pH** |
| --- | --- | --- | --- |
| Qingdao | 25.6 | 30.2 | 7.8 |
| Taizhou | 23.7 | 30.3 | 7.9 |
| Zhoushan | 23.0 | 30.0 | 7.5 |
| Haikou | 27.0 | 30.0 | 7.7 |
| Beihai | 28.0 | 30.0 | 7.6 |

pH, potential of hydrogen; PO_4_-P, phosphate phosphorus; S, Salinity; T, water temperature.
