# Supplementary material for: Integrative taxonomy and molecular phylogeny of three poorly known tintinnine ciliates, with the establishment of a new genus (Protista; Ciliophora; Oligotrichea)
Source: BMC Ecol Evol. 2021 Jun 9;21:115. doi: 10.1186/s12862-021-01831-8 (PMC8243829; doi:10.1186/s12862-021-01831-8)
Supplement: Supplementary file 2 — Additional file 2: Table S2. Morphometric data of Antetintinnopsis karajacensis comb. nov. from the literature matching our specimens in lorica shape. [file 12862_2021_1831_MOESM2_ESM.docx]

| **Lorica, length** | 100–120 | 100–120 | 111–172 | 144–152 | 60–80 | 184–230 | 113–127 | 120 |
| --- | --- | --- | --- | --- | --- | --- | --- | --- |
| **Lorica, width** | 40 | 40 | 55–64 | 45 | 30–30 | 76–95 | 46–57 | 55 |
| **OD** | 40 | 40 | 55–64 | 45 | 30–40 | 72–75 | 46–57 | 55 |
| **Line drawing** | Fig. S2a | Fig. S2b | Fig. S2c | Fig. S2d | Fig. S2e | Fig. S2f | Fig. S2g | Fig. S2h |
| **Data source** | [56] | [57] | [22] | [58] | [27] | [59] | [60] | [61] |

OD, opening diameter. Measurements in μm.
