# Supplementary material for: Integrative taxonomy and molecular phylogeny of three poorly known tintinnine ciliates, with the establishment of a new genus (Protista; Ciliophora; Oligotrichea)
Source: BMC Ecol Evol. 2021 Jun 9;21:115. doi: 10.1186/s12862-021-01831-8 (PMC8243829; doi:10.1186/s12862-021-01831-8)
Supplement: Supplementary file 3 — Additional file 3: Table S3. Morphology data of Antetintinnopsis gracilis comb. nov. from the literature matching our specimens in lorica shape. [file 12862_2021_1831_MOESM3_ESM.docx]

**Supplementary Table 3.** Morphology data of *Antetintinnopsis gracilis* comb. nov. from the literature matching our specimens in lorica shape.

| **Lorica, length** | 110–135 | 113 | 95 | 105–125 | 98–109 | 110 | 130 | 95–102 |
| --- | --- | --- | --- | --- | --- | --- | --- | --- |
| **Lorica, width** | 30–40 | 35 | 25 | - | 36–41 | - | 39 | 32–34 |
| **OD** | 30–40 | 25–45 | - | 28–34 | 30–35 | 33 | 39 | 29–30 |
| **Line drawing** | Fig. 11a | Fig. 11b | Fig. 11c | Fig. 11d | Fig. 11e | Fig. 11f | Fig. 11g | Fig. 11h |
| **Data source** | [26] | [63] | [64] | [22] | [65] | [60] | [66] | [67] |

OD, opening diameter. Measurements in μm. -, data absent.
