# Supplementary figures and images for: Integrative taxonomy and molecular phylogeny of three poorly known tintinnine ciliates, with the establishment of a new genus (Protista; Ciliophora; Oligotrichea)
Source: BMC Ecol Evol. 2021 Jun 9;21:115. doi: 10.1186/s12862-021-01831-8 (PMC8243829; doi:10.1186/s12862-021-01831-8)

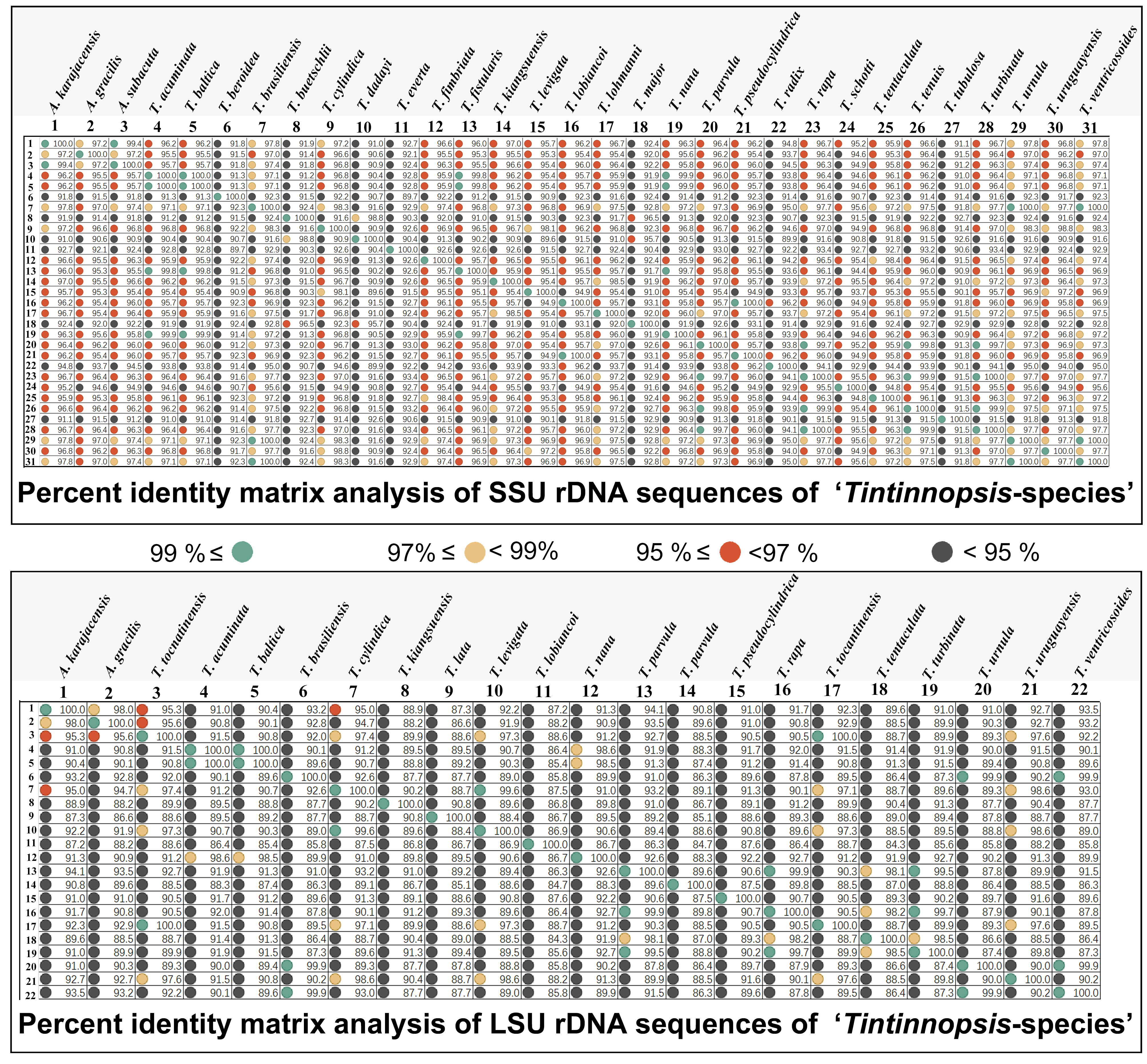

Supplement: Supplementary file 4 — Additional file 4: Fig. S1. Percent identity matrix of Antetintinnopsis karajacensis comb. nov. and A. gracilis comb. nov. with other Tintinnopsis-like species based on SSU and LSU DNA sequences. [file 12862_2021_1831_MOESM4_ESM.jpg]

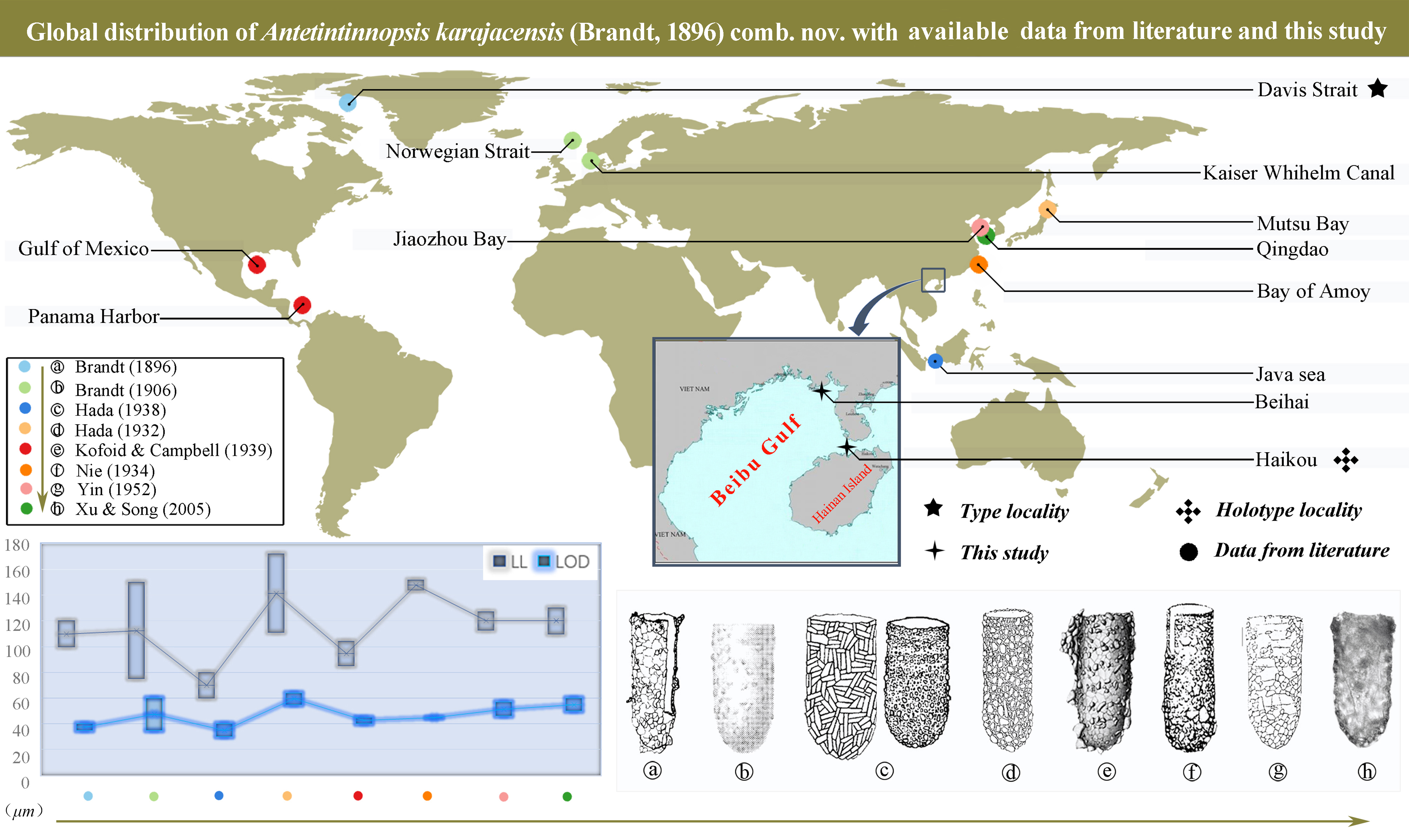

Supplement: Supplementary file 5 — Additional file 5: Fig. S2. Geographical distribution and morphological comparisons of different populations of Antetintinnopsis karajacensis comb. nov. based on the literatures and the present study. The vector maps were originally downloaded from the open-access website at http://mapsopensource.com/. All line drawings from open-access articles or books distributed under the terms of the Creative Commons Attribution License. LL, lorica length; LOD, lorica opening diameter. [file 12862_2021_1831_MOESM5_ESM.jpg]

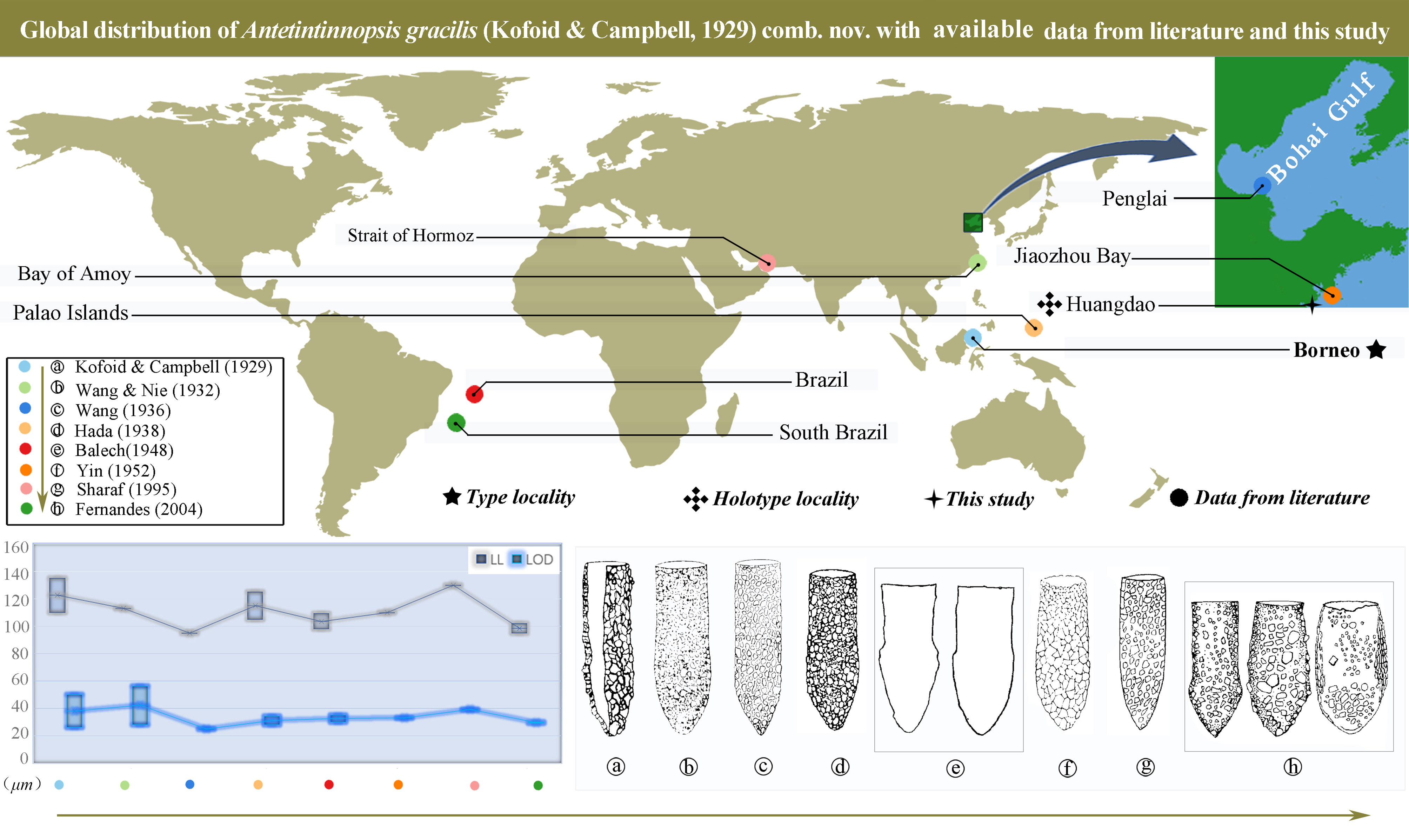

Supplement: Supplementary file 6 — Additional file 6: Fig. S3. Geographical distribution and morphological comparisons of different populations of Antetintinnopsis gracilis comb. nov. based on the literatures and the present study. The vector maps were originally downloaded from the open-access website at http://mapsopensource.com/. All line drawings from open-access articles or books distributed under the terms of the Creative Commons Attribution License. LL, lorica length; LOD, lorica opening diameter. [file 12862_2021_1831_MOESM6_ESM.jpg]
