# Supplementary material for: Integrative taxonomy and molecular phylogeny of three poorly known tintinnine ciliates, with the establishment of a new genus (Protista; Ciliophora; Oligotrichea)
Source: BMC Ecol Evol. 2021 Jun 9;21:115. doi: 10.1186/s12862-021-01831-8 (PMC8243829; doi:10.1186/s12862-021-01831-8)
Supplement: Supplementary file 7 — Additional file 7: Table S4. Characterization of the datasets and evolutionary models used for Bayesian analyses. [file 12862_2021_1831_MOESM7_ESM.docx]

| Dataset | Molecular marker | No. of taxa | No. of Chars | Evolutionary model | A | C | G | T | [AC] | [AG] | [AT] | [CG] | [CT] | [GT] | I | Γ |
| --- | --- | --- | --- | --- | --- | --- | --- | --- | --- | --- | --- | --- | --- | --- | --- | --- |
| #1 | SSU rDNA | 107 | 1705 | GTR+I+Γ | 0.2423 | 0.2178 | 0.2826 | 0.2574 | 1.3279 | 2.6858 | 1.6468 | 0.7526 | 4.7410 | 1.0000 | - | 0.6409 |
| #2 | LSU rDNA | 73 | 1292 | GTR+I+Γ | 0.2560 | 0.2187 | 0.2898 | 0.2354 | 1.1319 | 2.1499 | 0.9957 | 1.0395 | 4.2726 | 1.0000 | - | 0.9577 |

The best fitting evolutionary models were selected for each molecular marker separately under the Akaike information criterion in MrModeltest. A, C, G, T, base frequencies; [AC], [AG], [AT], [CG], [CT], [GT], rate substitution matrices; I, proportion of invariable sites; Γ, gamma distribution shape parameter.
